# Trophic ecology of groundfishes in nearshore areas of the Gulf of Maine

**DOI:** 10.1111/jfb.16026

**Published:** 2024-12-09

**Authors:** Landon P. Falke, Brian E. Smith, Stacy Rowe, Rebecca J. Peters, Timothy F. Sheehan

**Affiliations:** ^1^ Azura Consulting LLC Contractor for Northeast Fisheries Science Center, National Marine Fisheries Service, National Oceanic and Atmospheric Administration Woods Hole Massachusetts USA; ^2^ Northeast Fisheries Science Center, National Marine Fisheries Service, National Oceanic and Atmospheric Administration Woods Hole Massachusetts USA; ^3^ Maine Department of Marine Resources West Boothbay Harbor Maine USA

**Keywords:** climate impacts, diet information, prey availability, US northeast continental shelf

## Abstract

Ecosystem management requires an integrated understanding of ecological interactions. In the Gulf of Maine (GoM), trophic information pertaining to commercially important groundfishes and nearshore prey communities is lacking. We characterized nearshore communities and groundfish diets using data collected from nearshore surveys (864 bottom trawls and 3638 stomach samples of six groundfish species) conducted biannually (spring and fall) in Midcoast Maine and Penobscot Bay from 2012 to 2022. Groundfish diets were dominated by some of the most available nearshore prey (gadiform and clupeiform fishes and pandalid and crangonid shrimps). Shifts in relative prey availability over environmental gradients (e.g., depth and position along the coast), across seasons, and over years corresponded with parallel patterns in prey contributions to groundfish diets in specific predator–prey interactions. Negative trends in the relative availability and diet occurrence of signature GoM prey taxa (Northern shrimp *Pandalus borealis*, Atlantic herring *Clupea harengus*, and euphausiids) indicate that broader ecosystem changes, such as steady rises in water temperature and shifts in species distributions, are impacting nearshore trophic dynamics in the GoM. These observations provide timely information on mechanisms that underlie groundfish productivity and warrant inclusion of nearshore trophic dynamics in relevant ecosystem models.

## 1 INTRODUCTION

In contrast to offshore marine ecosystems, nearshore ecosystems are defined by tidally mixed currents, stronger freshwater interactions, and other abiotic characteristics (e.g., shallower depths and altered temperature regimes), which combine to provide unique functions for a variety of species. Many commercially and ecologically important species use nearshore environments at some point in their development for feeding (e.g., groundfishes; McDermott et al., 2015), reproduction (e.g., marine herrings; Lazzari & Stevenson, 1992), or migratory movements (e.g., diadromous fishes; Stevens et al., 2021). Understanding how broadly distributed species use and interact within nearshore systems is critical to developing effective ecosystem‐based management strategies. In particular, trophic dynamics are a primary regulator of fish production and an important consideration for ecosystem approaches to fisheries management (Gaichas et al., 2012; Link, 2010). Nearshore prey availability could play a pivotal role in the productivity of depleted groundfish stocks, emphasizing the need to address gaps in trophic information pertaining to groundfish in nearshore environments.

For centuries, groundfishes (i.e., fishes that dwell on or near the ocean floor) have supported substantial fisheries in the North Atlantic, but stocks have greatly diminished over time due to a combination of overfishing and oceanographic changes (Acheson & Gardner, 2014; Halliday & Pinhorn, 2009). In continental shelf ecosystems, groundfish can dominate biomass and regulate ecosystem function as generalist consumers of high amounts of prey and as prey themselves (Bowman & Michaels, 1984; Garrison & Link, 2000a; Rowe & Smith, 2022). Groundfish depletion can lead to pervasive, long‐term changes in trophic structure and ecosystem function (Choi et al., 2004; Frank et al., 2005), including replacement by crustaceans and other invertebrates (Howarth et al., 2014). In the US northeast continental shelf, groundfish trophic dynamics have been studied extensively to inform fisheries management, yet broad‐scale assessments have been limited to offshore environments (Garrison & Link, 2000a; Link & Almeida, 2000; Smith & Link, 2010). In contrast, groundfish trophic dynamics in nearshore environments have been largely overlooked, yet this information could be used to identify key prey resources, evaluate drivers of predator–prey population dynamics, and inform ecosystem approaches to fisheries management (Bax, 1998, Cury et al., 2005, Longo 2015). For instance, widespread efforts to restore diadromous fish communities could enhance the recovery of groundfish stocks by increasing cross‐ecosystem subsidies in coastal marine food webs (Ames & Lichter, 2013; Dias et al., 2019). This hypothesis has sparked recent interest in the contribution of diadromous fishes to groundfish diets (McDermott et al., 2015; Willis et al., 2017). However, the relative contributions of nearshore prey to groundfish diets and how it varies within and among predator species and with spatial and temporal factors are poorly understood, especially in the context of potential changes in nearshore trophic structure over time.

Diets of generalist predators in complex food webs are a response to relative prey availability in the local environment and mechanisms that influence predator–prey overlap in space and time (Baudrot et al., 2016; Chesson, 1984; Fryxell & Lundberg, 1994). Species composition in nearshore communities can be determined primarily by interactions between species traits (e.g., thermal preferences), bottom temperature, water column depth, and position along the coast (Jordaan et al., 2010; Zhang et al., 2011). Thus, these factors might explain spatial variation in groundfish foraging opportunities. Trophic interactions can also fundamentally change with temporal changes in species abundance or distribution. For instance, changes in the relative distribution of subarctic and mid‐Atlantic species in response to rapidly warming ocean conditions have led to long‐term, pervasive changes in trophic structure in the US northeast continental shelf (Friedland et al., 2019; Han et al., 2021). Responses of groundfish diets to declines and subsequent increases in diadromous fish production following watershed alteration and restoration are unclear. How generalist predators respond to changes in prey availability can depend on predator or prey traits (Kalinoski & DeLong, 2016; Preston et al., 2018; Schmidt et al., 2012). For instance, body size and gape morphology can determine the relative diet contributions of piscine prey in marine groundfish predators (Scharf et al., 2000). Thus, spatial or temporal changes in prey availability likely have differential effects on predators with different feeding habits (e.g., piscivores and invertivores), emphasizing the importance of multispecies trophic studies. Understanding how predator–prey interactions respond to ecosystem changes and its context dependencies (e.g., pertaining to nearshore systems and species‐specific responses) is an important component of the ecosystem knowledge needed to effectively manage groundfish and their prey populations.

The present study examines groundfish trophic ecology in nearshore areas of the Gulf of Maine (GoM) using data collected over an 11‐year period (2012–2022) aboard the Maine‐New Hampshire Inshore Trawl Survey (MEDMR, 2023). We combined survey data with stomach content sampling of six commercially and ecologically important groundfish species that use GoM nearshore habitats for feeding: silver hake *Merluccius bilinearis* (Mitchill 1814), red hake *Urophycis chuss* (Walbaum 1792), white hake *Urophycis tenuis* (Mitchill 1814), monkfish *Lophius americanus* Valenciennes 1837, Atlantic spiny dogfish *Squalus acanthias* L. 1758, and Atlantic cod *Gadus morhua* L. 1758. Our main objective was to address the basic need for trophic information pertaining to groundfishes in nearshore GoM environments and thereby identify important nearshore prey resources. To further characterize nearshore trophic ecology, we examined how nearshore prey availability, predator–prey size relationships, and prey contributions in groundfish diets vary over environmental gradients (depth, temperature, and longitudinal position along the coast) by season and across years. We also examined how the focal groundfish species differ with respect to diet and its relationship to nearshore prey availability. As a result, our study provides comprehensive insight into spatial and temporal processes that influence the foraging opportunities and potential productivity of groundfishes in the GoM.

## 2 MATERIALS AND METHODS

### 2.1 Data collection

#### 2.1.1 Study design

**FIGURE 1:**
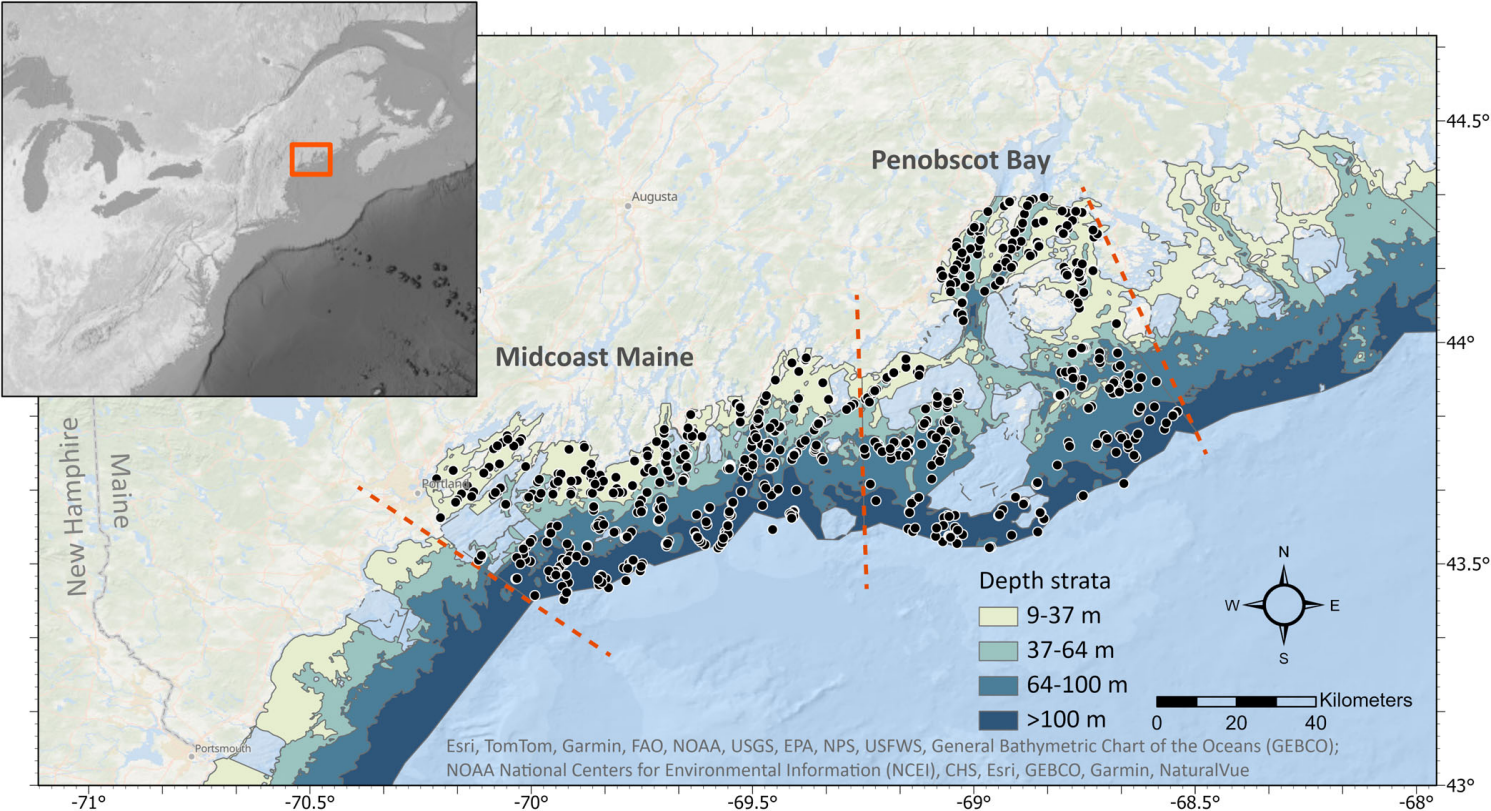
Map of study area showing all tow locations (black dots; *n* = 864), boundaries of the two longitudinal survey regions (red dashed lines), and depth strata. Blank areas within the survey regions (e.g., non‐filled portions near the center of Penobscot Bay) were identified as non‐towable areas due to rough bottom and were excluded from the survey design. Top left panel shows the study location (outlined in red) relative to the US northeast continental shelf.

Sampling was conducted aboard the Maine‐New Hampshire Inshore Trawl Survey from 2012 to 2022 in the nearshore GoM (Figure 1). The MENH Inshore Trawl Survey is a resource assessment survey that was established by the Maine Department of Marine Resources in 2000 to provide standardized monitoring in the nearshore GoM (MEDMR, 2023). This survey is conducted biannually (spring and fall) and uses a stratified random design that spans nearshore marine waters (within 22‐km maximum distance from the coastline) from New Hampshire to the eastern coastal region of Maine (MEDMR, 2023). The stratified design includes five longitudinal strata (i.e., regions) and four depth strata (9–37, 37–64, 64–100, and >100 m; MEDMR, 2023). The main purpose of the original stomach‐sampling efforts was to evaluate specifically the contributions of diadromous fishes (e.g., alosines) in groundfish diets (McDermott et al., 2015). The original study therefore focused on two of the five regions, Midcoast Maine and Penobscot Bay (located near the mouths of the Kennebec and Penobscot rivers, respectively; Figure 1), given their contemporary river herring populations and history of river restoration (McDermott et al., 2015). The present study is a continuation of this original study and focuses on the same two regions, but aims to characterize the trophic ecology of groundfishes more broadly.

Bottom trawls were conducted during daylight hours (7:00 a.m.–6:00 p.m.) using a commercial fishing vessel (the F.V. *Robert Michael*) equipped with a modified shrimp net (5 cm number 24 polyethylene mesh with 2.5‐cm mesh line on the cod end) designed to fish for near‐bottom dwelling species without targeting specific components (MEDMR, 2023). The spring surveys for Midcoast Maine and Penobscot Bay were conducted in mid‐to‐late May, and the fall surveys were conducted in early‐to‐mid October. Random stations were selected by plotting 1‐NM^2^ grids within strata, assigning each grid a unique call number, then using a random number generator. When habitat was deemed unsuitable for a bottom trawl based on prior experience or local knowledge (e.g., insufficient distance of suitable bottom, conflicts with fixed gear), a new random station was chosen within the same stratum, or the station was omitted if reassignment was impracticable. A target of 20–25 stations per region and per season was selected for sampling, with the number of tows per depth stratum apportioned based on its total area (~1 station for every 40 NM^2^). The target for tow duration was 20 min at a speed of 2.5 knots to cover roughly 0.8 NM. A conductivity, temperature, depth sensor was used to record salinity, temperature, and depth at the end of each tow. Latitude and longitude were recorded at the start and end of each tow.

#### 2.1.2 Tow catch processing

Organisms in each haul were identified to species, enumerated, weighed, and measured for length with subsampling procedures. When abundance of a certain species greatly exceeded 100 individuals, the total sample was homogenized (to avoid size‐related bias of hand‐selecting individuals), and ~100 individuals were removed as a subsample. All fish in subsamples were measured for total centerline length to the nearest whole centimeter. The aggregate weight of subsamples was used to estimate the total number caught and number of individuals at each centimeter increment. Abundance and weights of shrimp and euphausiids were estimated by species from 1‐kg homogenized subsamples when their collective mass greatly exceeded this amount in a given tow. Species abundances and masses in the catch were expanded based on a standard sampling effort of 20 min when tow duration was shorter than this time.

#### 2.1.3 Stomach sampling

Stomachs were sampled from *M. bilinearis*, *U. chuss*, *U. tenuis*, *L. americanus*, *S. acanthias*, and *G. morhua*. These six species are consistently present in the MENH Inshore Trawl Survey and represent potential predators of piscine prey. Therefore, they were selected for sampling in the original study that focused on the contributions of diadromous fishes in groundfish diets (McDermott et al., 2015). Stomach sampling was conducted at all biannual survey periods from 2012 to 2022, except in the Kennebec region in spring of 2012, the Penobscot region in fall of 2013, or in any region and season in 2020. We aimed to collect approximately 150 stomachs over each 5‐day survey leg (i.e., per region–season combination), portioned opportunistically across tows, depth strata, species, and the available size range, with minimum lengths of 15 cm for *L*. *americanus* and 20 cm for the other five predators. We avoided sampling fish with obvious signs of regurgitation (digested prey in mouth, flaccid stomach, or stomach protruding from mouth) or net feeding (fresh or actively moving prey in mouth or oesophagus). Each sampled fish was measured for total centerline length, and the whole stomach was removed and preserved with 95% ethanol on board the vessel. Stomachs were examined back in the laboratory using microscopy, and prey were identified to the lowest possible taxonomic level. Total mass (to the nearest 0.001 g) of all consumed prey within a stomach and individual lengths (to the nearest 1 mm) of intact piscine prey were recorded.

### 2.2 Data analysis

#### 2.2.1 Bottom temperature

We explored variation in bottom water temperature in relation to season, depth, position along the coast (longitude in our study), and year using non‐parametric analyses. Differences in bottom temperature between seasons (all years combined) were evaluated using permutational one‐way ANOVA. Given the significant differences in temperature between seasons, correlations between bottom temperature and continuous variables (depth, longitude, and year) were examined separately by season using Spearman rank correlation. For these Spearman correlations, scatterplots fit with locally estimated scatterplot smoothing (LOESS) were used to visualize the correlations and assess monotonicity. The “coin” package (Hothorn et al., 2008) in the statistical software R (R Core Team, 2023) was used to perform one‐way permutational ANOVA.

#### 2.2.2 Nearshore community structure

We used canonical correspondence analysis (CCA; ter Braak, 1986) to identify the primary drivers of community structure in the nearshore study area. The response matrix consisted of the proportional mass of each species within each tow. Rare species, when not sampled effectively, can bias the results of CCA (Borcard et al., 2018). To focus this analysis on common taxa, we aggregated taxa that were present in <5% of tows into higher classifications (e.g., Bivalvia, Teleostei, Cephalopoda, Invertebrata) until all remaining taxa were present in ≥5% of tows. CCA was conducted with a forward‐selection procedure (“ordistep” function in the “vegan” package in R; Oksanen et al., 2019) to determine the simplest combination of explanatory variables. Using forward selection, variables that explained a higher amount of variation in the response matrix were prioritized in order in the final CCA model, and variables that did not explain a significant amount of variation were dropped. AIC was used to rank the models in each successive step. CCA was performed on tow‐level proportional mass data within each season separately to account for potential seasonal dependencies of species–environment relationships and of changes in composition across years. The explanatory matrix consisted of continuous variables for year, depth, longitude, and bottom temperature. Year was treated as continuous to evaluate patterns across the study period rather than year‐to‐year variation. All continuous explanatory variables were scaled and centered to account for differences in measurement units. We calculated variance inflation factors (VIF) using the “vif.cca” function to assess collinearity among explanatory variables in the final models, and accepted the final model if all VIFs were <5. We performed permutation tests with 999 permutations using the “anova.cca” function to determine whether the final model and individual terms explained more variation in the response matrix than expected by random chance.

For each season, we further evaluated relationships between the proportional mass of individual species in catch (i.e., a univariate response) and the continuous explanatory variables using Spearman rank correlation. For Spearman correlations, scatterplots fit with LOESS were used to visualize the correlations and assess monotonicity. We also used permutational one‐way ANOVA to test for an overall difference in mean proportional mass between seasons for each species. These sets of tests were focused on the groundfish predators and taxa that were confirmed as prey for groundfishes.

#### 2.2.3 Groundfish diets

Diet composition for each predator species was summarized by frequency of occurrence (i.e., the proportion of predator stomachs in which a certain prey taxon was observed) and proportional mass (i.e., the proportion of mass of a certain prey taxon relative to the total mass of all prey in a given predator stomach). For each predator species, frequency occurrence (FO) of prey type *k* was calculated as follows:

$${\mathrm{FO}}_{k}=\frac{p_{k}}{N}$$

where *N* is the total number of stomachs sampled, and *p*_*k*_ is the number of stomachs containing prey type *k*. For proportional mass, we calculated an overall weighted mean across tows accounting for clustered samples by weighting the data by the number of individuals of each predator species per tow (see Buckel et al., 1999; Cochran, 1977). The weighted mean proportional mass (PM) of prey type *k* in a given predator species was calculated as follows:

$${\mathrm{PM}}_{k}=\frac{\sum_{i=1}^{n}W_{i}\frac{q_{\mathrm{ik}}}{q_{i}}}{\sum W_{i}}$$

where *n* is the number of tows in which the predator was sampled, *W*_*i*_ is the number of individuals of the predator in tow *i*, *q*_*i*_ is the total mass of all prey types in stomach samples of the predator in tow *i*, and *q*_*ik*_ is the mass of prey type *k* in stomach samples of the predator in tow *i*. To evaluate the adequacy of stomach sample sizes, cumulative prey diversity curves were plotted using the Shannon–Weiner diversity index as a function of randomized stomach number for each predator–season combination. Asymptotic stabilization of each cumulative prey curve was visually assessed to determine if the number of stomachs needed to adequately characterize diets was met.

For each predator species, we performed CCA with a forward‐selection procedure to determine the simplest combination of variables that significantly influence diet composition, similar to the approach we used to analyse nearshore community structure. The diet CCA included tow‐level proportional mass of prey taxa (highest resolution classifications) in stomachs as the response matrix. We limited the dataset to only include tows with ≥4 stomachs (species‐specific) to avoid inflation of variance from lower sample sizes. We aggregated prey taxa that were present in <5% of tows into higher classifications until all remaining taxa were present in ≥5% of tows. The response matrix consisted of tow‐level data from both seasons combined because power (i.e., sample size of tows with ≥4 stomachs) was relatively limited compared to the nearshore community structure CCA. Thus, the diet CCAs included a categorical variable for season along with the same continuous explanatory variables that were used in the nearshore community structure CCA (year, depth, longitude, and bottom temperature). The continuous explanatory variables were scaled and centered to account for differences in measurement units. Similarly, we calculated VIFs for explanatory variables in the final model (accepted if all VIF <5) and performed permutation tests (999 permutations) to determine significance of the final model and terms.

We further evaluated relationships between the diet contribution (tow‐level proportional mass) of each individual prey taxon and the continuous explanatory variables using Spearman rank correlation. Scatterplots were fit with LOESS to visualize correlations and assess monotonicity. Seasonal differences in mean diet contributions were tested using permutation one‐way ANOVA. These tests were performed on all prey taxa included in the CCA for each predator species.

#### 2.2.4 Prey occurrence probability

To supplement the diet composition analyses described above, we used Bayesian logistic regression with a Markov chain Monte‐Carlo (MCMC) algorithm to estimate the effects of predator traits, environmental variables, temporal factors, and relative prey availability on the probability of prey‐specific occurrence in stomach contents. We focused this analysis on a subset of focal prey taxa using taxonomic groupings that best aligned with the taxonomic resolution of identifications in stomach contents. The focal prey taxa were Alosinae (i.e., shads; subfamily in the herring family Clupeidae), Atlantic herring *Clupea harengus* L. 1758, *M. bilinearis*, Euphausiidae (krill), and northern shrimp *Pandalus borealis*. We selected these prey taxa because they represented a substantial portion of nearshore community composition and stomach contents of the groundfish predators, and evaluating their occurrence in stomach contents provides insight into nearshore trophic dynamics in the context of broader ecosystem changes (e.g., watershed restoration and climate impacts) that might be influencing the abundance or distribution of these prey.

For each prey taxon, we first fit a Bayesian logistic regression with the binomial response of prey occurrence in a stomach sample at the individual predator level and fixed effects for predator species, predator size (total center length), tow depth, season, longitude, bottom temperature, and year. This model structure was used to supplement the tow‐level diet analysis by modeling prey‐specific occurrence at the individual predator level and estimating effects of the spatial and temporal variables, while also accounting for effects of predator size. To make the intercept term more interpretable, we scaled and centered predator size, depth, longitude, and bottom temperature, and converted year to an integer from 0 to 9 (2012–2022, excluding 2020). To then estimate the effects of relative prey availability on individual‐level prey occurrence directly, we fit a separate model for each prey taxon with the same binomial response of prey occurrence, fixed effects for predator species identity and predator size, and a new fixed effect for relative prey availability (the proportional mass of the prey taxon in the tow in which the predator was sampled). This second model structure was used to evaluate feeding responses to changes in prey availability and how these responses differ among predator species, while also accounting for predator size. The dataset consisted of a large number of tows (*n* = 525 tows with stomach samples), sample sizes within tows were small (mean <7 samples per tow), and intra‐tow correlation in prey‐specific occurrences appeared minimal based on data exploration. We explored models with a random effect of tow identity, but these multilevel models exhibited divergent transitions. Therefore, the model was simplified, and individuals sampled in the same tow were assumed to be independent observations. The models were fit with the “brms” package (“brms” translates R code to the software STAN; Bürkner, 2017) in R using diffuse normal priors and a logit link function. Posterior samples were drawn from four MCMC chains, with 50,000 iterations per chain, warmup of 20,000, and a thinning interval of five. Chain convergence for each parameter was assessed with trace plots and accepted when the potential scale reduction factor was under 1.1 (Gelman et al., 2013). We evaluated posterior estimates and 95% credible intervals for each parameter, where a 95% credible interval overlapping with zero was interpreted as insufficient evidence of a meaningful effect on the probability of prey‐specific occurrence in predator stomachs. For plotting marginal effects of relative prey availability on predicted probability of prey occurrence in groundfish stomach contents, predicted values were drawn from the fit models using the “tidybayes” package (Kay, 2023) in R. All analyses were performed using R version 4.2.2 (R Core Team, 2023).

## 3 RESULTS

### 3.1 Bottom temperature

Mean bottom temperature was roughly twofold higher in fall (x̄_FALL_ = 11.08°C, SD_FALL_ = 1.91°C) compared to spring (x̄_SPRING_ = 5.72°C, SD_SPRING_ = 1.17°C; permutational ANOVA: Z = −24.85, *p* < 0.001). Bottom temperature was negatively correlated with depth in both seasons, but the decrease was steeper in fall compared to spring (Figure S1). Bottom temperature had non‐linear relationships with longitude but increased overall from west to east, especially in fall (Figure S1). Over the study period (2012–2022), bottom temperatures marginally increased due to higher‐than‐average temperatures in the most recent years, but the correlation was not significant (Figure S1).

### 3.2 Nearshore community structure

**FIGURE 2:**
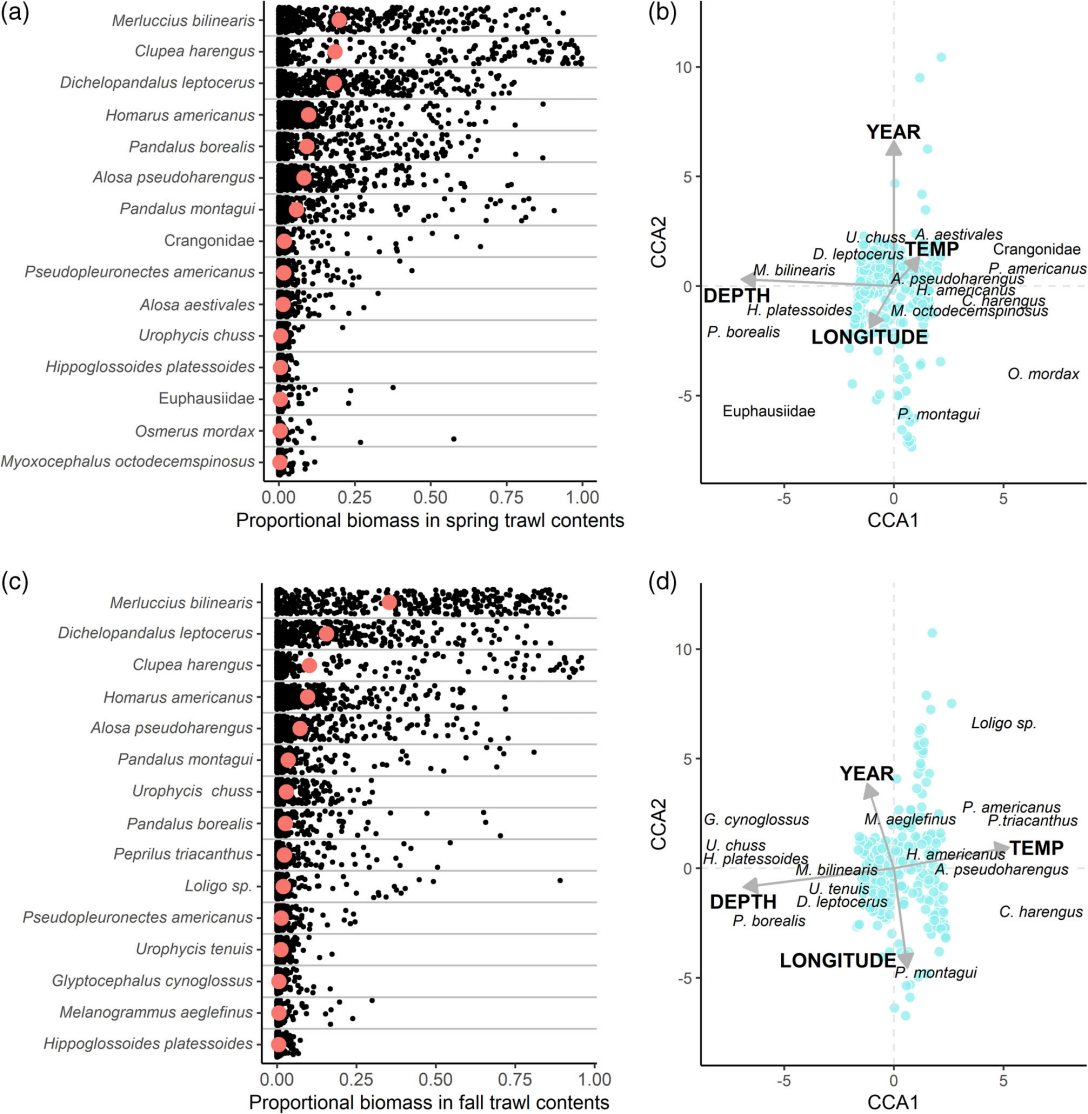
Proportional biomasses of the 15 most common taxa in the nearshore survey (Midcoast Maine and Penobscot Bay regions from 2012 to 2022) and ordination biplots depicting the associated canonical correspondence analysis (CCA) results for spring (a and b) and fall (c and d). In panels (a) and (c), black dots represent proportional mass in individual tows, and the red points represent the mean proportional mass in that season overall (years and regions combined). Blue points in panels (b) and (d) represent individual tows and their similarity in composition by species proportional mass. CCA vectors are labeled with their corresponding explanatory variable, where “TEMP” represents bottom temperature at the tow location. In the ordination plots, species centroids for the 15 most common taxa are labeled (name is centered on the point). To aid visualization, CCA vectors and species centroids were scaled by a factor of 6, and some species centroids were adjusted minimally while maintaining their relative position (i.e., to avoid overlapping labels).

In both spring and fall seasons, proportional catch biomass was dominated by *M. bilinearis*, *C. harengus*, pandalid shrimp (*Dichelopandalus leptocerus*, *P. borealis*, and *Pandalus montagui*), alewife *Alosa pseudoharengus* (Wilson 1811), and American lobster *Homarus americanus* (Figure 2). However, there were several significant differences in relative catch composition between seasons, including greater proportional masses of *C*. *harengus*, *P*. *borealis*, blueback herring *Alosa aestivalis* (Mitchill 1814), crangonid shrimp, and *P*. *montagui* in the spring. In contrast, the proportional masses of American butterfish *Peprilus triacanthus* (Peck 1804), longfin squid (*Loligo* sp.), *L*. *americanus*, *U*. *chuss*, *U*. *tenuis*, *M*. *bilinearis*, and *S*. *acanthias* were significantly greater in the fall. Means and SDs of proportional masses in tow catches by season and permutational ANOVA results for each taxon analysed are provided in Table S1.

The highest amount of explained variation in species composition among tows was attributed to depth, followed by year, longitude, and bottom temperature, representing the order of explanatory variables in the final model for each season (Figure 2; see supplemental materials for more details on CCA of nearshore community structure). The proportional mass of nearly all common taxa showed strong relationships with depth (Figures S2 and S3). Notably, taxa that showed seasonally consistent decreases in proportional mass in catch with increasing depth (deeper tows) included crangonids, *P*. *triacanthus*, *C*. *harengus*, American shad *Alosa sapidissima* (Wilson 1811), and *A*. *aestivalis*. *A. pseudoharengus* decreased with increasing depth in fall but not in spring when the relationship appeared non‐linear. In contrast, taxa that showed seasonally consistent increases in proportional mass with increasing depth included euphausiids, *P*. *borealis*, fourbeard rockling *Enchelyopus cimbrius* (L. 1766), *L*. *americanus*, *U*. *chuss*, *U*. *tenuis*, *M*. *bilinearis*, and *S*. *acanthias*. *P. montagui* and *D*. *leptocerus* showed a non‐linear relationship with depth, peaking in intermediate depths. Proportional masses of certain taxa showed directional changes over the 11‐year study period, whereas others only showed year‐to‐year variation with no overall correlation (Figures S4 and S5). *P. montagui* and *P*. *borealis* showed clear annual declines in proportional masses in both seasons, *C. harengus* declined annually in fall (but showed no annual change in spring), and euphausiids declined annually in spring (but showed no annual change in fall). In contrast, *U*. *chuss* and *L*. *americanus* increased annually in both seasons, *M*. *bilinearis* and *S*. *acanthias* increased annually in fall (but no annual change in spring), and *D*. *leptocerus* increased annually in spring (but no annual change in fall). Longitudinal variation in catch composition included strong west‐to‐east correlations for several taxa (Figures S6 and S7). Proportional masses of *D*. *leptocerus* and *P*. *montagui* increased from west to east, whereas *A*. *aestivalis*, *A*. *sapidissima*, and *S*. *acanthias* decreased. Certain correlations with longitude were non‐linear (e.g., *P*. *borealis* and *M*. *bilinearis*) or differed in shape by season (e.g., *A*. *pseudoharengus*). Proportional masses of most taxa in catch were significantly correlated with bottom temperature (Figures S8 and S9). In both seasons, proportional mass of *P*. *borealis* decreased with increasing temperature. Four of the focal groundfish predators (*U*. *chuss*, *U*. *tenuis*, *M*. *bilinearis*, and *S*. *acanthias*) also showed decreases with increasing temperatures but only in fall. In contrast, *P*. *triacanthus* and *H*. *americanus* increased with increasing temperature in both seasons, and *C*. *harengus* increased with temperature in fall.

The body size distributions of the focal groundfish predators and piscine prey taxa varied by season and depth (Figures S10 and S11). Larger predators were generally more common in fall and in deeper locations (Figure S10). The size of piscine prey (e.g., *A*. *aestivalis*, *A*. *pseudoharengus*, *A*. *sapidissima*, *C*. *harengus*, and *P*. *triacanthus*) also increased with increasing depth, especially in fall (Figure S11).

### 3.3 Groundfish diets

**FIGURE 3:**
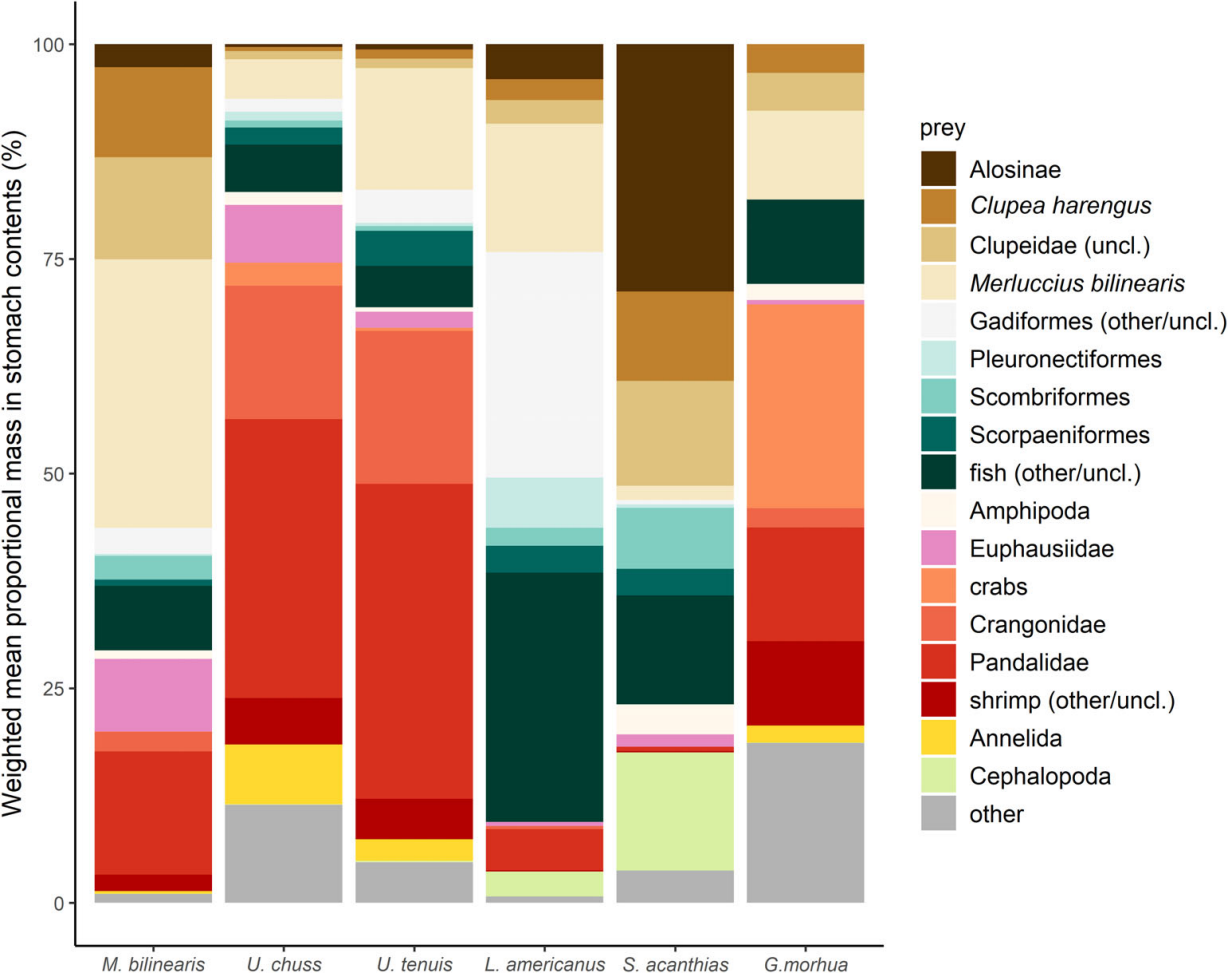
Weighted mean proportional mass of prey taxa in stomach contents of *Merluccius bilinearis* (*n* = 1364), *Urophycis chuss* (*n* = 611), *Urophycis tenuis* (*n* = 947), *Lophius americanus* (*n* = 514), *Squalus acanthias* (*n* = 150), and *Gadus morhua* (*n* = 52) sampled in the nearshore Gulf of Maine. Certain prey categories are aggregations of other identifiable taxa and/or unclassified (uncl.) taxa within a higher‐level classification. The “other” category includes other invertebrate taxa and unidentifiable animal remains. Fish prey are represented in the nine uppermost prey categories using a brown to blue gradient beginning with Alosinae and ending with the “fish (other/uncl.)” category. Diet metrics for all prey taxa (best possible resolution identifications) are provided in Table S3.

**FIGURE 4:**
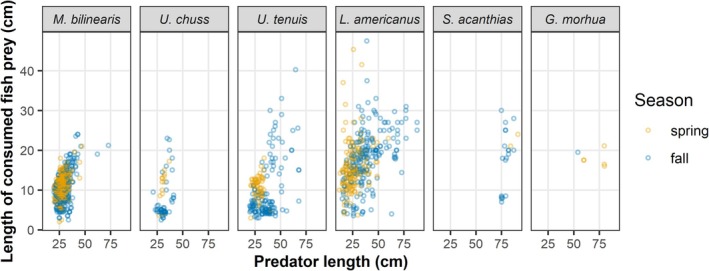
Sizes of consumed fish prey by corresponding predator size in stomach contents of *Merluccius bilinearis*, *Urophycis chuss*, *Urophycis tenuis*, *Lophius americanus*, *Squalus acanthias*, and *Gadus morhua* in the nearshore Gulf of Maine. Points represent all intact and measurable fish prey observed in each predator and are colored by the sampling season (spring in orange and fall in blue).

We sampled a total of 3638 groundfish stomachs, consisting of 1364 *M*. *bilinearis*, 611 *U*. *chuss*, 947 *U*. *tenuis*, 514 *L*. *americanus*, 150 *S*. *acanthias*, and 52 *G*. *morhua* (Table S2). Diet composition differed considerably among predator species, including the relative diet contributions of fish prey compared to invertebrate prey (Figure 3; Table S3) and the size range of consumed fish prey (Figure 4). Shannon diversity of stomach contents indicated that overall sample sizes were adequate to characterize the diet of each predator except *G. morhua* (Figure S12).

#### 3.3.1 M. bilinearis

**FIGURE 5:**
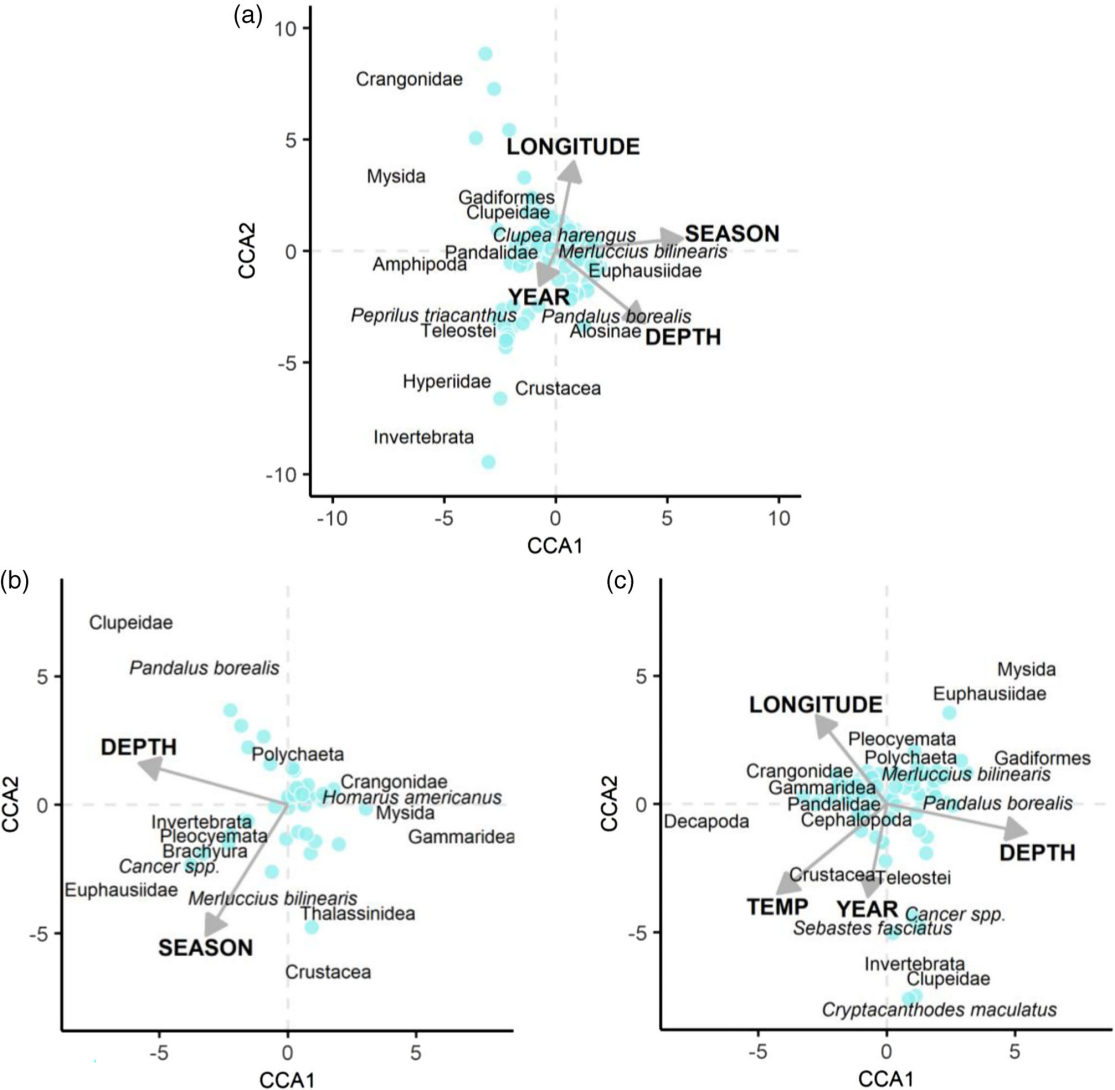
Ordination biplots displaying results of canonical correspondence analysis (CCA) on diet composition (proportional mass of prey taxa) in *Merluccius bilinearis* (a), *Urophycis chuss* (b), and *Urophycis tenuis* (c). Biplots for the other three predators are not shown because CCA models did not explain significant variation in their diet composition (*Lophius americanus* and *Squalus acanthias*) or sample sizes were insufficient (*Gadus morhua*). Blue points represent individual tows (with ≥4 stomach samples) and their similarity in diet composition by tow‐level proportional mass of prey in stomach contents. CCA vectors are labeled with their corresponding explanatory variable, where “TEMP” represents bottom temperature at the tow location. To aid visualization, CCA vectors and species centroids were scaled by a factor of 6. Species labels are centered on their respective centroid, and some were adjusted minimally while maintaining their relative position (i.e., to avoid overlapping labels). Additionally, species centroids near the origin (0, 0), which have low correlation with the CCA axes, are not labeled.

Stomach contents of *M*. *bilinearis* consisted mostly of conspecifics (i.e., *M*. *bilinearis* cannibalism), clupeiforms (*C*. *harengus* and unclassified clupeids), pandalid shrimp, and euphausiids (Figure 3; Table S3). Variation in *M*. *bilinearis* diet composition was explained by season (DF = 1, 137; F = 5.73; *p* < 0.001), depth (DF = 1, 137; F = 2.34; *p* = 0.003), year (DF = 1, 137; F = 1.85; *p* = 0.013), and longitude (DF = 1, 137; F = 1.76; *p* = 0.023), representing the order of explanatory variables in the final CCA model (DF = 4, 137; F = 2.92; *p* < 0.001). The final model explained 7.9% of variation in diet composition, with the first and second CCA axes representing 4.2% and 1.9%, respectively (Figure 5a). *M. bilinearis* consumed relatively more (tow‐level proportional mass) conspecifics and euphausiids in the spring, whereas they consumed more clupeids, crangonids, pandalids, *P*. *triacanthus*, and unclassified teleosts in the fall (Table S4). With increasing depth, *M*. *bilinearis* consumed relatively more euphausiids, hyperiid amphipods, and conspecifics and less crangonid shrimp, mysids, and pandalids (Figure S13). Consumption of crangonids, mysids, and *P*. *triacanthus* was positively correlated with bottom temperature, whereas consumption of alosines (i.e., Alosinae), euphausiids, and conspecifics was negatively correlated with temperature (Figure S13). Correlations by longitude included an increase in crangonids and decreases in alosines, *P*. *triacanthus*, and unclassified teleosts from west to east (Figure S13). Relative consumptions of clupeids and hyperiids in *M*. *bilinearis* diets were negatively correlated with year, whereas crangonids and unclassified invertebrates were positively correlated with year (Figure S13).

#### 3.3.2 U. chuss

Stomach contents of *U*. *chuss* consisted mostly of shrimp (pandalids, crangonids, and unclassified shrimps), polychaetes, and euphausiids (Figure 3; Table S3). The relatively small contribution of piscine prey in *U*. *chuss* diets consisted mostly of *M*. *bilinearis* and unclassified fishes. Variation in *U*. *chuss* diet composition was mostly explained by depth (DF = 1, 42; F = 2.27; *p* < 0.001) and season (DF = 1, 42; F = 1.88; *p* = 0.015), representing the order of explanatory variables in the final CCA model (DF = 2, 42; F = 2.07; *p* < 0.001). The final model explained 9.0% of variation in diet composition, with the first and second CCA axes representing 5.0% and 4.0%, respectively (Figure 5b). Diets of *U*. *chuss* included relatively more (tow‐level proportional mass) euphausiids in the spring and more crangonids in the fall (Table S4). With increasing depth, *U*. *chuss* consumed more euphausiids and *P*. *borealis* and less crangonids (Figure S14). Consumption of crangonids was positively correlated with bottom temperature, whereas consumption of euphausiids was negatively correlated with temperature (Figure S14). Correlations by longitude included decreases in consumption of *M*. *bilinearis* and unclassified teleosts from west to east (Figure S14). The relative consumption of *P*. *borealis* in *U*. *chuss* diets was negatively correlated with year (Figure S14).

#### 3.3.3 U. tenuis

Stomach contents of *U*. *tenuis* consisted mostly of pandalids, crangonids, and *M*. *bilinearis*, although a variety of piscine taxa contributed to their diets overall (Figure 3; Table S3). Variation in *U*. *tenuis* diet composition among tows was explained by depth (DF = 1, 76; F = 3.48; *p* < 0.001), bottom temperature (DF = 1, 76; F = 2.64; *p* = 0.002), longitude (DF = 1, 76; F = 1.95; *p* = 0.011), and year (DF = 1, 76; F = 2.07; *p* = 0.017), representing the order of explanatory variables in the final CCA model (DF = 4, 76; F = 2.53; *p* < 0.001). The final model explained 11.8% of variation in diet composition, with the first and second CCA axes representing 4.4% and 3.1%, respectively (Figure 5c). Diets of *U*. *tenuis* included relatively more (tow‐level proportional mass) euphausiids, *M*. *bilinearis*, mysids, and unclassified shrimps in the spring and more pandalids in the fall (Table S4). With increasing depth, *U*. *tenuis* consumed more crabs (*Cancer* spp.), gadiforms, *P*. *borealis*, *M*. *bilinearis*, and unclassified teleosts, and less crangonids, decapods, and pandalids (Figure S15). Consumption of crangonids, unclassified decapods, and pandalids was positively correlated with bottom temperature, whereas consumption of euphausiids and *M*. *bilinearis* was negatively correlated with temperature (Figure S15). Correlations by longitude included increased consumption of crangonids, pandalids, and polychaetes and decreased consumption of wrymouth *Cryptacanthodes maculatus* Storer 1839, Acadian redfish *Sebastes fasciatus* Storer 1854, and unclassified teleosts from west to east (Figure S15). The relative consumption of *P*. *borealis* and unclassified shrimps in *U*. *tenuis* diets were negatively correlated with year (Figure S15).

#### 3.3.4 L. americanus

Stomach contents of *L*. *americanus* were dominated by piscine prey, including a variety of taxa but mostly consisting of gadiforms (*M*. *bilinearis*, *E. cimbrius*, *Urophycis* spp.), clupeiforms (alosines, *C*. *harengus*), pleuronectiforms, and unclassified teleosts (Figure 3; Table S3). The relatively small contribution of invertebrates in *L*. *americanus* diets consisted mostly of pandalid shrimp and cephalopods. None of the CCA terms explained more variation in *L*. *americanus* diet composition than expected by random chance (*n* = 34 tows with ≥4 stomach samples); thus, the final CCA model for *L*. *americanus* included only the intercept. However, three seasonal differences in *L*. *americanus* diets were significant, with greater consumption (tow‐level proportional mass) of *E*. *cimbrius*, pandalids, and unclassified crustaceans in spring compared to fall (Table S4). Two correlations by longitude were also significant, with greater relative consumption of snake blenny *Ophidion barbatum* L. 1758 in more eastern longitudes and Atlantic mackerel *Scomber scombrus* L. 1758 in more western longitudes (Figure S16). Consumption of pandalids was negatively correlated with temperature, whereas consumption of *U*. *chuss* was positively correlated with temperature (Figure S16).

#### 3.3.5 S. acanthias

Stomach contents of *S*. *acanthias* consisted mostly of alosines and other clupeids, which combined for over half of *S*. *acanthias* diet by mean proportional mass (Figure 3; Table S3). Cephalopods, unclassified teleosts, *S*. *scombrus*, *C*. *maculatus*, and hyperiid amphipods were the next largest identified contributors in *S*. *acanthias* diets. *S. acanthias* had the highest rate of empty stomachs (35.33%) of the six focal predators (Table S3). None of the CCA terms explained more variation in *S*. *acanthias* diet composition than expected by random chance (*n* = 16 tows with ≥4 stomach samples), and there were no correlations between tow‐level proportional masses of prey and the explanatory variables. Seasonal differences in *S. acanthias* diets were not evaluated due to their low sample size in spring (Figure S12).

#### 3.3.6 G. morhua

Stomach contents of *G*. *morhua* consisted mostly of crabs (*Cancer* spp. and unclassified crabs), pandalids, and other decapods (Figure 3; Table S3). The next largest identified contributors in *G*. *morhua* diets were *M*. *bilinearis*, clupeiforms, and *H. americanus*. The sample size of *G*. *morhua* stomachs was not sufficient to assess potential sources of diet variation (e.g., depth and longitude) using CCA or Spearman rank correlation.

### 3.4 Prey‐specific occurrence probability in stomach contents

The effects of predator traits, environmental covariates, and temporal factors on the occurrence probabilities of five prey taxa (Alosinae, *C*. *harengus*, *M*. *bilinearis*, *P*. *borealis*, Euphausiidae) in groundfish stomachs were significant in many cases (Table S5). For all five prey taxa, occurrence probabilities differed by predator species. Predator size had significant positive effects on occurrence probabilities of each of the three piscine prey taxa and significant negative effects on Euphausiidae. However, predator size was not meaningful in the *P*. *borealis* model (i.e., the 95% credible interval overlapped with zero). Season had significant effects on occurrence probability of four of the five prey taxa, with higher probabilities of alosines, *C*. *harengus*, *M*. *bilinearis*, and Euphausiidae in spring diets. With increasing depth, probabilities of alosines and *C*. *harengus* in diets decreased. In contrast, probabilities of Euphausiidae, *P*. *borealis*, and *M*. *bilinearis* increased with depth. Longitude (increasing west to east) had meaningful effects on occurrence probabilities of only *M*. *bilinearis* (negative). Occurrence probabilities of *C*. *harengus*, *M*. *bilinearis*, *P*. *borealis*, and Euphausiidae in diets each decreased across years. The posterior mean effect estimate of year was most negative in *P*. *borealis* (−0.40, 95% CI = [−0.50, −0.30]), followed by *C*. *harengus* (−0.19, 95% CI = [−0.28, −0.11]), Euphausiidae (−0.11, 95% CI = [−0.15, −0.06]), and M. bilinearis (−0.06, 95% CI = [−0.10, −0.02]), but year was not meaningful in the Alosinae model (0.06, 95% CI = [−0.07, 0.19]). Bottom temperature was not meaningful in any of the five prey‐occurrence models and was therefore excluded in the final models. Posterior mean effect estimates and 95% credible intervals for all fixed effects in the five prey‐occurrence models are compiled in Table S5.

**FIGURE 6:**
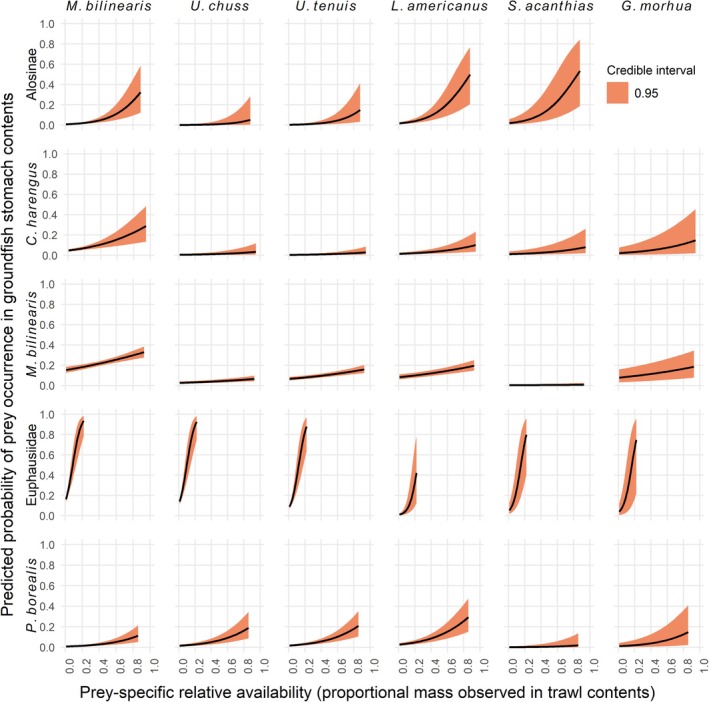
Marginal effects of prey relative availability (proportion) on predicted probability of prey occurrence in groundfish stomach contents (proportion) for different predator–prey combinations in the nearshore Gulf of Maine. Each column represents a focal groundfish predator species (i.e., a categorical fixed effect): *Merluccius bilinearis*, *Urophycis chuss*, *Urophycis tenuis*, *Lophius americanus*, *Squalus acanthias*, and *Gadus morhua*. Each row represents a focal prey taxon (i.e., a separate Bayesian logistic regression model), including *Clupea harengus* and *Pandalus borealis*. Predicted values of prey occurrence probability were not drawn for values of relative prey availability that exceeded the maximum observed proportional mass in the nearshore trawls (e.g., maximum proportional mass of euphausiids observed in trawls contents = 0.23). Predator size was included as a continuous fixed effect in each model and set equal to the overall mean for calculation of marginal effects. The red band displays the 95% credible interval. *G. morhua* was excluded from the Alosinae model because there were no confirmed observations of this prey taxon in its stomach contents.

Occurrence probabilities of the five prey taxa were each positively influenced by relative prey availability (i.e., prey‐specific proportional mass in the tow from which the predator was sampled). Posterior mean effect estimates of prey availability varied considerably among the prey taxa and was highest in Euphausiidae (20.59, 95% CI = [14.13, 27.38]), followed by Alosinae (4.71, 95% CI = [3.13, 6.20]), *P*. *borealis* (3.21, 95% CI = [2.22, 4.17]), *C*. *harengus* (2.22, 95% CI = [1.18, 3.17]), and *M*. *bilinearis* (1.09, 95% CI = [0.68, 1.50]). The marginal effects of prey availability on predicted probability of prey occurrence in stomach contents varied in slope among predator–prey combinations (Figure 6).

## 4 DISCUSSION

The productivity of commercially important groundfish depends on nearshore prey availability for foraging during development, making an understanding of nearshore trophic dynamics critical to the management of depleted groundfish stocks. Stomach contents of groundfish predators sampled from nearshore areas in the GoM consisted of a wide taxonomic breadth of prey, but they were generally dominated by some of the most abundant nearshore prey (gadiform and clupeiform fishes, pandalid and crangonid shrimps). This supports previous characterization of these groundfish species as generalist, opportunistic feeders based on studies in adjacent offshore environments (Garrison & Link, 2000a, 2000b; Link & Garrison, 2002a, 2002b; Smith & Link, 2010). Opportunistic feeding was further supported by the increased probability of prey occurrence in stomach contents with increasing availability of the prey in the local environment. As a primary determinant of generalist predator diets, changes in relative prey availability can drive diet variation across ontogenetic stages (Sánchez‐Hernández et al., 2017), environmental gradients (Terraube & Arroyo, 2011), and a wide range of temporal scales (Howells et al., 2017; Mills et al., 2020; Sigler et al., 2009). In many cases, relative prey availability and their contributions to groundfish diets showed parallel patterns (i.e., same directions of correlation) by depth, temperature, longitudinal position along the coast, season, or across years. For instance, clupeids (alosines and *C*. *harengus*) were more likely to occur in stomachs sampled in shallower depths, whereas *M*. *bilinearis*, *P*. *borealis*, and euphausiids were more likely to occur in stomachs sampled in deeper areas, matching patterns by depth in their availability (proportional biomass in trawls). As another key example, the consumption of *C*. *harengus*, *P*. *borealis*, and euphausiids declined across years, coinciding with observed declines in their availability in our study as well as previously documented declines in their abundance in the North Atlantic (Edwards et al., 2021; Lasley‐Rasher et al., 2015; Richards & Hunter, 2021). These patterns represent meaningful spatial and temporal changes in the topology of nearshore food webs. However, not all groundfish diet responses were well explained by variation in prey availability (e.g., flat slopes between prey availability and probability of prey occurrence in stomachs), highlighting the need to consider predator‐ and prey‐specific characteristics rather than prey availability alone. In particular, the role of thermal affinities in mediating trophic responses of groundfish predator–prey interactions to ocean warming warrants consideration (Selden et al., 2018). The multispecies characterization of groundfish trophic ecology in nearshore environments that our study provides can support broader ecosystem science and management in the GoM, as this ecosystem continues to change rapidly.

Given the influence of prey availability on diets of generalist predators, groundfish trophic ecology in nearshore systems is better understood in the context of nearshore community structure. Depth explained the greatest amount of variation in nearshore community composition of our explanatory variables, with proportional biomass of species showing negative (e.g., *C*. *harengus* and crangonid shrimp), positive (e.g., *M*. *bilinearis* and *P*. *borealis*), or unimodal (e.g., *A*. *pseudoharengus* and *D*. *leptocerus*) relationships with depth. These species‐depth associations are certainly driven by corresponding changes in other environmental variables, rather than depth alone, such as temperature, pressure, light, and resource availability. Significant variation in nearshore community composition was also explained by longitudinal position along the coast. Several environmental factors that vary along coastlines can influence species distributions, such as substrate composition (Pihl & Wennhage, 2005), bathymetry (Malek et al., 2014), influences of freshwater drainages (e.g., temperature and salinity gradients; Abookire et al., 2000), and oceanographic processes (e.g., coastal currents; Watson et al., 2011). Bottom temperature also explained significant variation in nearshore community structure even after accounting for the higher amounts of variation explained by depth, longitude, and year. Temperature is a key determinant of geographic distributions of benthic marine organisms (Belanger et al., 2012), and most taxa observed in the nearshore surveys had some correlation with bottom temperature. Understanding species' direct relationships with bottom temperature alone is challenging due to relationships between temperature and other key variables (e.g., depth and longitude in our study). A more robust understanding of nearshore trophic dynamics could be obtained by characterizing relationships of nearshore species with variables not directly examined in our study (e.g., light, salinity, substrate, and primary production) and conducting a more detailed investigation relating temperature variability to species abundance while controlling key covariates (e.g., using marginal effects analysis). Nonetheless, our results support studies that identify interactions between depth, position along the coast, and temperature as primary determinants of nearshore community structure in the GoM (Jordaan et al., 2010; Zhang et al., 2011). In turn, these factors are important determinants of spatial variation in groundfish foraging opportunities in nearshore environments.

Temporal community dynamics also dictate groundfish foraging opportunities in nearshore environments. Temperature at depth varies seasonally on the US northeast continental shelf, which can drive seasonal changes in habitat use of thermally sensitive species (Methratta & Link, 2006; Murawski & Finn, 1988). The observed seasonality of nearshore community composition and species‐depth associations in our study is probably related to a combination of seasonal changes in temperature regimes and species physiology and phenology (e.g., thermal sensitivity, seasonal migration, growth, and reproduction). Additionally, the US northeast continental shelf ecosystem is experiencing long‐term shifts in temperatures regimes, including rapid rates of annual warming in response to climate impacts (Friedland & Hare, 2007), which are expected to alter the distribution and abundance of thermally sensitive species in the GoM (reviewed by Pershing et al., 2021). For instance, the GoM is expected to see decreases in several signature subarctic species (e.g., the copepod *Calanus finmarchicus*, northern krill *Meganyctiphanes norvegica*, *P*. *borealis*, *C*. *harengus*, *G*. *morhua*) and increases in mid‐Atlantic species (e.g., longfin squid *Doryteuthis pealeii*, S. *scombrus*, *M*. *bilinearis*; Lasley‐Rasher et al., 2015; Pershing et al., 2021). Bottom temperatures in the nearshore surveys only marginally increased across years (non‐significant), but nearshore community composition significantly changed over the study period (2012–2022). This included declines in the relative biomass of certain prey resources (*C*. *harengus*, *P*. *borealis*, *P*. *montagui*, and euphausiids) along with increases in *D*. *leptocerus* and four of the focal groundfish predators (*L*. *americanus*, *S*. *acanthias*, *U*. *chuss*, and *M*. *bilinearis*). Although natural cycles in species biomass occurring over decadal time scales could explain long‐term variation in trophic interactions (e.g., Glaser, 2010), the trophic dynamics of nearshore areas in the GoM is certainly influenced by broader oceanic changes and geographic shifts in species distributions. For example, the declines observed in our study and more broadly (Lasley‐Rasher et al., 2015, Pershing et al., 2021) for *C*. *harengus*, *P*. *borealis*, and euphausiids were mirrored in groundfish stomachs. Expanding long‐term monitoring of nearshore trophic interactions can complement the broad‐scale monitoring efforts that focus on offshore environments (Link & Almeida, 2000), providing greater spatial coverage and temporal overlap of trophic information to help predict population‐ and ecosystem‐level responses to climate impacts in this region.

The contribution of diadromous fishes to groundfish diets could be a factor in enhancing the resiliency of groundfish populations to long‐term changes in nearshore prey resources. We confirmed groundfish predation on three anadromous species in the subfamily Alosinae: *A. pseudoharengus*, *A*. *aestivalis*, and *A*. *sapidissima*. Alosine migrations to and from the mouths of freshwater drainages provide seasonal pulses of high‐lipid prey resources in nearshore areas (Ames, 2004; Ames & Lichter, 2013; McDermott et al., 2015; Willis et al., 2017). Alosines contributed to diets of each focal predator, except *G. morhua* (possibly due to its low sample size), and were more likely to occur in larger individuals and predator species that primarily consumed fish. Previous studies have confirmed *G*. *morhua* predation on alosines in nearshore habitats (McDermott et al., 2015, Willis et al., 2017), and it has been hypothesized that alosines have historically controlled the success of *G*. *morhua* year classes in the GoM (Ames, 2004; Ames & Lichter, 2013). Alosine contributions to groundfish diets appear negligible at broad scales (e.g., US northeast continental shelf; Garrison & Link, 2000a, Smith et al., 2007) but are substantially greater when considered at spatial and temporal scales relevant to their overlap with groundfish predators (McDermott et al., 2015). Digestion and morphological similarities with other clupeids (e.g., *C*. *harengus*) make it difficult to obtain precise estimates of alosine contributions to groundfish diets using stomach content analysis, and integrating DNA‐based diet assessments would further clarify these interactions. Their contributions to groundfish diets should also be considered in the context of the study area (i.e., close proximity to the Kennebec and Penobscot rivers) and historical trends in their populations. Historically, *Alosa* spp. were abundant in the northeastern US (Saunders et al., 2006) but have diminished due to impacts of dams on accessibility to spawning and nursery habitats (Hall et al., 2011, 2012; Limburg & Waldman, 2009). The reduction in ecosystem services, including their role as prey in marine food webs, has been attributed to these declines (reviewed by Hare et al., 2021, Ouellet et al., 2022). The Kennebec, Penobscot, and other nearby watersheds (e.g., St. Croix River in Maine‐New Brunswick) have the potential to contribute >50 million alosines into the GoM annually, but current production is much lower (Hall et al., 2012). Based on recent increases in alosine abundance in Penobscot Bay following major dam removals (Stevens et al., 2024), restoring watershed connectivity is a potential solution to enhancing the diadromous fish prey base in nearshore environments. However, the lack of any significant increase in alosine prey availability or consumption by groundfishes over our study period suggests that their production in freshwater systems is still a limiting factor.

How groundfish diets respond to spatial and temporal changes in prey availability depends on their diet configuration, which differed considerably among predators in our study. Piscine prey dominated the diets of *L*. *americanus*, *S*. *acanthias*, and *M*. *bilinearis*, whereas the diets of gadids (*U*. *chuss*, *U*. *tenuis*, and *G*. *morhua*) were composed mostly of macroinvertebrate prey. Differences in diet composition and prey size selection among sympatric predators can be associated with differences in foraging strategies, anatomical features, mobility, or space use (Falke et al., 2020; Michalko & Pekár, 2016; Schmitz, 2017). For example, the barbels of gadids, jaw protusion of *S. acanthias*, and gape size and lure of *L. americanus* enable feeding strategies suited for different prey types. Habitat use also differed among predator species, with *L*. *americanus* and *S*. *acanthias* being less common in shallow tows compared to the more widespread *M*. *bilinearis*, *U*. *chuss*, and *U*. *tenuis*. Our analysis of diet variation within species suggested that diets of *M*. *bilinearis*, *U*. *chuss*, and *U*. *tenuis* are correlated with spatial and temporal variation in prey resources. The amount of diet variation explained by CCA for these three groundfish was low (7.9%–11.8%) but comparable to those reported by Byron and Link (2010; ~6%–13% in US northeast groundfish) and Jaworski and Ragnarsson (2006; ~6%–16% in Icelandic groundfish). Similar to our findings, Link et al. (2002) reported up to 11.7% of explained diet variation in North Atlantic groundfish with multiple species that had no significant amount of explained variation in diet despite sufficient sample sizes. Our explanatory variables did not explain a significant amount of diet variation in *L*. *americanus* and *S*. *acanthias*. The unexplained diet variation could be related to several factors, including selective feeding behaviors, individual‐level processes, or other variables not included in our analysis. For example, trophic niche variation and stochastic processes at the individual level, including size‐based feeding, can explain high intraspecific variation in fish stomach contents (Beyer, 1998; Cachera et al., 2017), but our diet CCA models analysed tow‐level data. Substrate is another potential source of variation in nearshore community composition (Zhang et al., 2011) and might also contribute to diet variation in groundfish. Our sampling was biased to low‐complexity habitats where bottom trawls were feasible and cannot be extended to high‐complexity areas where species composition and trophic interactions might differ. The insufficient sample size of *G*. *morhua* is at least partly due to their preference for complex bottom habitats (Methratta & Link, 2007). However, Willis et al. (2017) characterized *G*. *morhua* diets in the nearshore GoM and found seasonal and spatial differences related to concurrent variation in the relative availability of piscine and macroinvertebrate prey.

Predator–prey size relationships and its variation by depth and season have a considerable role in groundfish trophic ecology in nearshore environments. The size ranges of groundfishes and certain piscine prey (e.g., *C*. *harengus*, alosines, *P*. *triacanthus*) decreased from deeper to more shallow habitats and were relatively small in spring compared to fall. Given the important role of predator–prey size relationships on trophic interactions, this size structure of nearshore communities at least partly explains some of the groundfish diet variation by depth and season. Size‐related habitat partitioning and associated diet shifts have been documented in each of the focal predator species (Garrison & Link, 2000a, 2000b; Hanson, 2011; Methratta & Link, 2007). Diets generally shift from macroinvertebrate‐dominated feeding in smaller size classes to piscine‐dominated feeding in larger size classes. These ontogenetic diets shifts may be less pronounced overall in predators that grow to smaller adult sizes (e.g., *U*. *chuss*; Sedberry, 1983) or may occur in relatively young stages in predators with large gapes (e.g., *L*. *americanus*; Johnson et al., 2008). Our evaluation of size‐related diet shifts was limited because large individuals of each predator species were not common in the nearshore surveys, and small individuals (i.e., <20 cm) were not included in our sampling design. With the exception of *S*. *acanthias*, the size ranges of groundfishes that we sampled are small to intermediate compared to offshore studies (Garrison & Link, 2000a; Link & Garrison, 2002a). Nonetheless, the diet observations supported expectations of size‐based feeding, with more and larger piscine prey in larger predators. Groundfish responses to changes in prey resources may therefore differ at the individual level in relation to size‐based trophic ecology.

Selective prey preferences can explain diet responses that contradict availability‐based feeding in generalist predators (Whitney et al., 2018). Relationships between predicted probability of prey occurrence in groundfish stomach and prey‐specific relative availability differed among predator–prey combinations in our logistic regression models. At one extreme, occurrence probability of euphausiids in stomach contents increased steeply with small increases in their relative availability, suggesting a strong preference for this prey, when available. In contrast, occurrence probability of *M*. *bilinearis* in stomach contents showed only small increases with large increases in their relative availability. Some degree of prey selectivity in generalist predators may be explained by differences in capturability or energetic profitability (e.g., lipid content and energy density) among prey resources (Fryxell & Lundberg, 1994; Schmidt et al., 2012; Stephens & Krebs, 1986). For example, the extremely low rates of predation on lobsters (*H*. *americanus*) and flatfishes (Pleuronectiformes), despite these taxa being among the largest sources of biomass in the surveys, could be related to their antipredator defenses (e.g., claws, camouflage). Given their dominant biomass in nearshore systems, the trophic role of *H*. *americanus* as prey for other predators in the GoM is a particularly important avenue for future research that would benefit from expanding nearshore trophic studies to include other benthivores. Selective feeding in *S*. *acanthias* has been observed previously (Overholtz et al., 2000; Spencer & Collie, 1996), and it likely explains the substantially higher proportions of clupeids and cephalopods but lower proportions of *M*. *bilinearis* in their diets compared to the other five predator species. Occurrence probability of *P*. *borealis* and alosines in stomach contents showed relatively steep increases with their availability compared to *M*. *bilinearis* and *C*. *harengus*, but shapes of these relationships depended on predator species. For instance, *U*. *chuss* and *U*. *tenuis* showed relatively little response to alosine availability compared to the other predators, whereas *U*. *chuss*, *U tenuis*, and *L*. *americanus* showed stronger responses to *P*. *borealis* availability compared to the other predators. Thus, changes in prey‐specific availability should affect groundfishes differentially based on their selective diet preferences.

Trophic information can be used for a broad range of fisheries management applications, such as creating prey abundance indices (Ng et al., 2021), estimating natural prey regulation (Smith & Smith, 2020), assessing community trends (Gaichas et al., 2024), or parameterizing multispecies stock assessment models (Trijoulet et al., 2020). Considering that many of the nearshore predator and prey species examined here have much broader distributions, integrating our work with broader‐scale studies (see Link & Almeida, 2000) would contribute to implementation of ecosystem‐based fisheries management in the Northwest Atlantic (Link et al., 2011; Pikitch et al., 2004). Our study can also inform management strategies at smaller scales of resource management, particularly for coastal fisheries in the GoM that are directly supported by groundfishes and certain prey species (e.g., *H*. *americanus*, *C*. *harengus*, *A*. *pseudoharengus*; Waller et al., 2023). Although the roles of predator and prey traits (e.g., thermal affinity, size, and behavior) should be considered, the dynamics of groundfish diets can be controlled largely by prey availability and have the potential to explain regional and annual patterns in groundfish productivity (e.g., Buchheister et al., 2015). Continued monitoring over time periods relevant to climate impacts, as well as broadening the scope of trophic studies by including disparate geographic areas, species, and trophic levels, can improve our understanding of spatial and temporal drivers of groundfish productivity. Information on nearshore trophic ecology is valuable for identifying sources of variability in groundfish productivity and should be incorporated in relevant models that support ecosystem approaches to fisheries management.

## AUTHOR CONTRIBUTIONS

Timothy F. Sheehan contributed to project administration and supervision of the research. Rebecca J. Peters led survey and data collection, with field support from Landon P. Falke and Stacy Rowe. Landon P. Falke and Timothy F. Sheehan conceptualized ideas for the manuscript with support from Brian E. Smith and Stacy Rowe. Landon P. Falke led data analyses and manuscript preparation. All authors reviewed and edited the manuscript.

## FUNDING INFORMATION

This study was funded by National Oceanic and Atmospheric Administration (NA22NMF4540361).

## Supporting information


**Data S1.** Supporting information.
