# Supplementary material for: Trophic ecology of groundfishes in nearshore areas of the Gulf of Maine
Source: J Fish Biol. 2024 Dec 9;106(4):1095–111. doi: 10.1111/jfb.16026 (PMC12038783; doi:10.1111/jfb.16026)
Supplement: Supplementary file 1 — Data S1. Supporting information. [file JFB-106-1095-s001.docx]

Supplementary Materials

**Trophic ecology of groundfishes in nearshore areas of the Gulf of Maine**

Landon P. Falke, Brian E. Smith, Stacy Rowe, Rebecca J. Peters, and Timothy F. Sheehan

*Canonical correspondence analysis of nearshore community structure* – Variation in species composition among spring tows was significantly explained by depth (DF = 1, 449; F = 53.69; P < 0.001), year (DF = 1, 449; F = 11.20; P < 0.001), longitude (DF = 1, 449; F = 8.43; P < 0.001), and bottom temperature (DF = 1, 449; F = 4.92; P < 0.001), representing the order of explanatory variables in the final spring model (DF = 4, 449; F = 19.56; P < 0.001). The final model explained 14.8% of variation in spring catch composition, with the first and second CCA axes representing 10.3% and 2.2%, respectively (Fig. 2B). The first CCA axis was strongly correlated with depth (biplot score = -0.99), and the second CCA axis was most strongly correlated with year (biplot score = 0.94).

Variation in species composition among fall tows was also significantly explained by depth (DF = 1, 405; F = 47.19; P < 0.001), year (DF = 1, 405; F = 8.19; P < 0.001), longitude (DF = 1, 405; F = 6.74; P < 0.001), and bottom temperature (DF = 1, 405; F = 3.01; P = 0.008), representing the order of explanatory variables in the final fall model (DF = 4, 405; F = 16.28; P < 0.001). The final model explained 13.9% of variation in fall catch composition, with the first and second CCA axes representing 10.4% and 2.1% of the explained variation, respectively (Fig. 2D). The first CCA axis was strongly correlated with depth (biplot score = -0.98) and bottom temperature (biplot score = 0.75), and the second CCA axis was mostly correlated with longitude (biplot score = 0.65) and year (biplot score = -0.55).


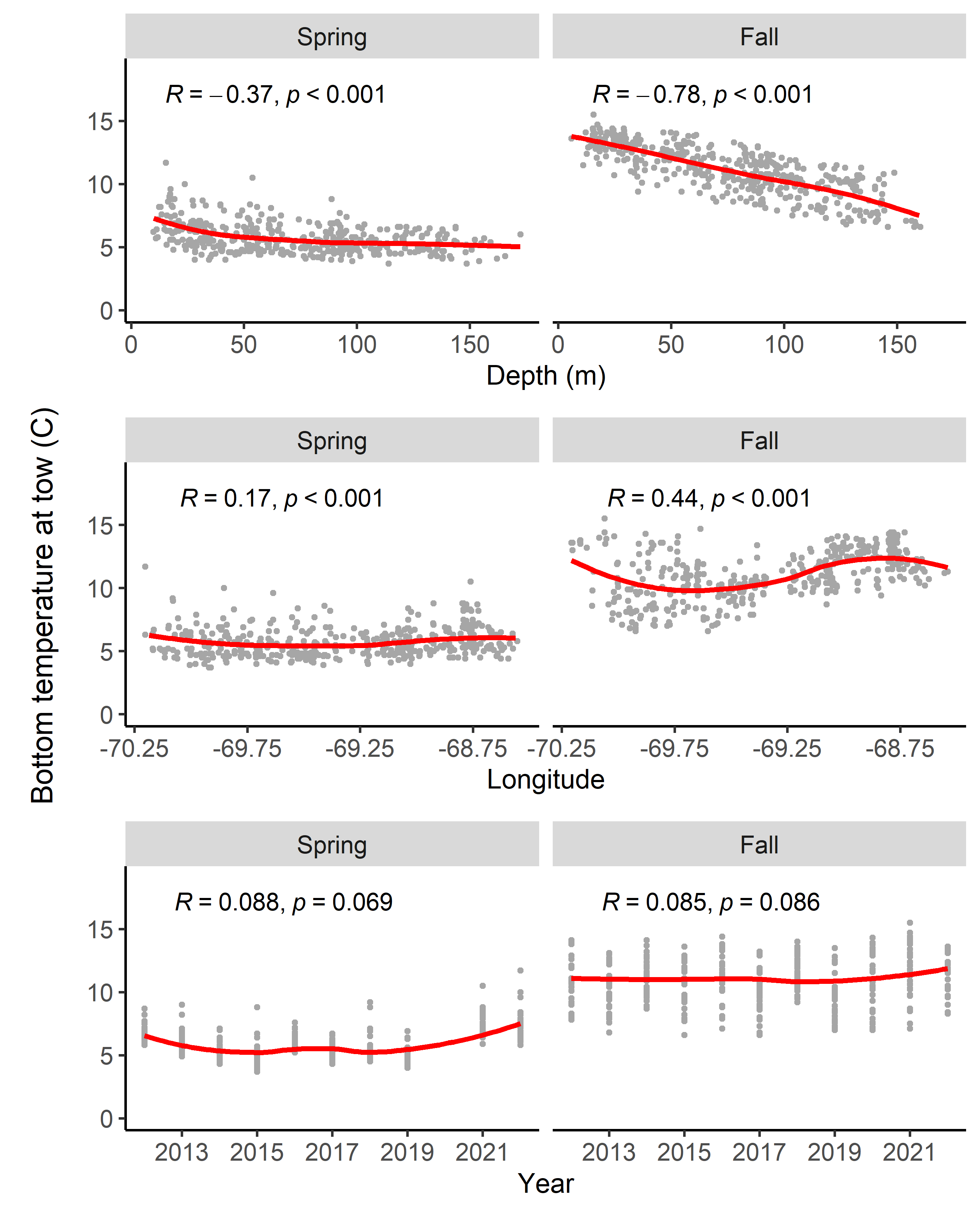


Figure S1. Scatter plots depicting relationships between bottom temperature and depth, longitude, and survey year in the nearshore Gulf of Maine. Results of Spearman rank correlation analysis are displayed in each plot. Gray points represent individual tows. Red lines show the fit of locally estimated scatterplot smoothing (LOESS). Statistical significance of a non-monotonic relationship should be considered a spurious correlation.

Table S1. Mean and standard deviation of proportional mass of taxa in the nearshore trawls (n = 864) and results of permutational one-way ANOVA between seasons.

| Taxon | x̄_SPRING_ | SD_SPRING_ | x̄_Fall_ | SD_Fall_ | Z | P.value |
| --- | --- | --- | --- | --- | --- | --- |
| *Alosa aestivalis* | 0.013 | 0.032 | 0.003 | 0.023 | 5.156 | <.001 |
| *Alosa pseudoharengus* | 0.083 | 0.124 | 0.073 | 0.131 | 1.154 | 0.248 |
| *Alosa sapidissima* | 0.001 | 0.003 | 0.001 | 0.008 | -0.592 | 0.554 |
| Brachyura (true crabs) | 0.005 | 0.018 | 0.003 | 0.013 | 2.038 | 0.042 |
| *Clupea harengus* | 0.185 | 0.309 | 0.102 | 0.234 | 4.34 | <.001 |
| Crangonidae | 0.018 | 0.068 | 0.005 | 0.046 | 3.328 | 0.001 |
| *Dichelopandalus leptocerus* | 0.182 | 0.193 | 0.156 | 0.184 | 2.023 | 0.043 |
| *Enchelyopus cimbrius* | 0.001 | 0.002 | 0.001 | 0.003 | -2.079 | 0.038 |
| Euphausiidae | 0.005 | 0.026 | 0.002 | 0.015 | 1.65 | 0.099 |
| *Gadus morhua* | 0.001 | 0.004 | <.001 | 0.001 | 1.81 | 0.07 |
| *Glyptocephalus cynoglossus* | 0.002 | 0.005 | 0.006 | 0.017 | -4.975 | <.001 |
| *Hippoglossoides platessoides* | 0.005 | 0.008 | 0.006 | 0.012 | -0.459 | 0.646 |
| *Homarus americanus* | 0.098 | 0.142 | 0.096 | 0.135 | 0.18 | 0.857 |
| *Illex* sp. | <.001 | <.001 | 0.003 | 0.007 | -8.647 | <.001 |
| *Loligo* sp. | 0.001 | 0.005 | 0.02 | 0.077 | -5.233 | <.001 |
| *Lophius americanus* | <.001 | 0.001 | 0.001 | 0.004 | -5.684 | <.001 |
| *Melanogrammus aeglefinus* | 0.001 | 0.004 | 0.006 | 0.028 | -4.047 | <.001 |
| *Merluccius bilinearis* | 0.199 | 0.23 | 0.354 | 0.281 | -8.556 | <.001 |
| *Pandalus borealis* | 0.092 | 0.163 | 0.026 | 0.079 | 7.241 | <.001 |
| *Pandalus montagui* | 0.058 | 0.148 | 0.035 | 0.107 | 2.541 | 0.011 |
| *Peprilus triacanthus* | 0.003 | 0.035 | 0.023 | 0.068 | -5.41 | <.001 |
| *Pseudopleuronectes americanus* | 0.016 | 0.045 | 0.013 | 0.031 | 0.992 | 0.321 |
| *Scomber scombrus* | <.001 | 0.001 | 0.003 | 0.011 | -5.017 | <.001 |
| *Sebastes fasciatus* | 0.001 | 0.005 | 0.004 | 0.012 | -4.5 | <.001 |
| *Squalus acanthias* | <.001 | <.001 | 0.001 | 0.003 | -3.724 | <.001 |
| *Tautogolabrus adspersus* | 0.001 | 0.004 | 0.003 | 0.03 | -1.471 | 0.141 |
| *Urophycis chuss* | 0.006 | 0.013 | 0.029 | 0.054 | -8.665 | <.001 |
| *Urophycis tenuis* | 0.001 | 0.002 | 0.013 | 0.017 | -12.805 | <.001 |

Table S2. Number of stomachs sampled from six groundfish predators in nearshore Gulf of Maine by season, region (i.e., longitudinal stratum), and year range.

| Predator taxon | Spring | | | | | | |  | Fall | | | | | | |  |
| --- | --- | --- | --- | --- | --- | --- | --- | --- | --- | --- | --- | --- | --- | --- | --- | --- |
|  | Midcoast Maine | | |  | Penobscot Bay | | |  | Midcoast Maine | | |  | Penobscot Bay | | |  |
|  | 2012-2015 | 2016-2018 | 2019-2022 |  | 2012-2015 | 2016-2018 | 2019-2022 |  | 2012-2015 | 2016-2018 | 2019-2022 |  | 2012-2015 | 2016-2018 | 2019-2022 | Total |
| *Merluccius bilinearis* | 79 | 118 | 157 |  | 134 | 101 | 188 |  | 126 | 86 | 89 |  | 62 | 98 | 126 | 1364 |
| *Urophycis chuss* | 15 | 27 | 52 |  | 15 | 40 | 50 |  | 94 | 45 | 60 |  | 53 | 78 | 82 | 611 |
| *Urophycis tenuis* | 63 | 55 | 42 |  | 82 | 88 | 66 |  | 95 | 77 | 80 |  | 90 | 114 | 95 | 947 |
| *Lophius americanus* | 14 | 58 | 26 |  | 35 | 50 | 39 |  | 40 | 74 | 39 |  | 31 | 54 | 54 | 514 |
| *Squalus acanthias* | 0 | 0 | 8 |  | 0 | 0 | 0 |  | 1 | 29 | 22 |  | 12 | 15 | 63 | 150 |
| *Gadus morhua* | 6 | 3 | 1 |  | 19 | 9 | 0 |  | 3 | 3 | 0 |  | 7 | 1 | 0 | 52 |

Table S3. Percent frequency of occurrence (FO) and mean proportional biomass (PM) of prey taxa in stomach contents of six groundfish predators in nearshore Gulf of Maine. Cells with a dash (-) indicate no observation of the prey taxon in the predator species. Cells with “*NA*” indicate that a calculation was not made or not applicable at this level.

|  | *Merluccius bilinearis* | |  | *Urophycis chuss* | |  | *Urophycis tenuis* | |  | *Lophius americanus* | |  | *Squalus acanthias* | |  | *Gadus morhua* | |
| --- | --- | --- | --- | --- | --- | --- | --- | --- | --- | --- | --- | --- | --- | --- | --- | --- | --- |
| Prey taxon | %FO | %PM |  | %FO | %PM |  | %FO | %PM |  | %FO | %PM |  | %FO | %PM |  | %FO | %PM |
| **Amphipoda** |  |  |  |  |  |  |  |  |  |  |  |  |  |  |  |  |  |
| Amphipoda | 0.07 | <0.01 |  | 0.16 | <0.01 |  | - | - |  | - | - |  | - | - |  | - | - |
| Caprellidae | 0.07 | <0.01 |  | 0.49 | 0.01 |  | - | - |  | - | - |  | 0.67 | <0.01 |  | - | - |
| Gammaridea | 1.10 | 0.10 |  | 6.55 | 1.43 |  | 4.75 | 0.49 |  | 0.19 | <0.01 |  | - | - |  | 5.77 | 1.86 |
| Hyperiidae | 3.08 | 0.88 |  | 1.64 | 0.08 |  | 1.16 | 0.01 |  | 0.19 | <0.01 |  | 3.33 | 3.50 |  | - | - |
| Total Amphipoda | *NA* | 0.98 |  | *NA* | 1.52 |  | *NA* | 0.50 |  | *NA* | <0.01 |  | *NA* | 3.50 |  | *NA* | 1.86 |
|  |  |  |  |  |  |  |  |  |  |  |  |  |  |  |  |  |  |
| **Annelida** |  |  |  |  |  |  |  |  |  |  |  |  |  |  |  |  |  |
| Polychaeta | 0.37 | 0.31 |  | 13.58 | 6.93 |  | 7.29 | 2.49 |  | 0.19 | <0.01 |  | 0.67 | 0.03 |  | 11.54 | 1.98 |
|  |  |  |  |  |  |  |  |  |  |  |  |  |  |  |  |  |  |
| **Argentiniformes** |  |  |  |  |  |  |  |  |  |  |  |  |  |  |  |  |  |
| Argentinidae | 0.07 | 0.19 |  | - | - |  | - | - |  | - | - |  | - | - |  | - | - |
|  |  |  |  |  |  |  |  |  |  |  |  |  |  |  |  |  |  |
| ***Cephalopoda*** |  |  |  |  |  |  |  |  |  |  |  |  |  |  |  |  |  |
| Cephalopoda | 0.07 | 0.02 |  | 0.49 | 0.07 |  | 0.74 | 0.20 |  | 1.55 | 1.29 |  | 6.67 | 6.30 |  | 1.92 | 0.03 |
| *Illex* sp. | - | - |  | - | - |  | - | - |  | 0.58 | 1.14 |  | 0.67 | 3.61 |  | - | - |
| *Loligo* sp. | - | - |  | - | - |  | - | - |  | 0.39 | 0.48 |  | 1.33 | 3.85 |  | - | - |
| Total Cephalopoda | *NA* | 0.02 |  | *NA* | 0.07 |  | *NA* | 0.20 |  | *NA* | 2.91 |  | *NA* | 13.76 |  | *NA* | 0.03 |
|  |  |  |  |  |  |  |  |  |  |  |  |  |  |  |  |  |  |
| **Clupeiformes** |  |  |  |  |  |  |  |  |  |  |  |  |  |  |  |  |  |
| *Alosa aestivalis* | 0.44 | 1.25 |  | - | - |  | 0.11 | 0.05 |  | - | - |  | - | - |  | - | - |
| *Alosa pseudoharengus* | 0.59 | 0.98 |  | - | - |  | 0.11 | 0.35 |  | 2.52 | 2.23 |  | - | - |  | - | - |
| *Alosa sapidissima* | - | - |  | - | - |  | 0.11 | 0.14 |  | - | - |  | - | - |  | - | - |
| Alosinae | 0.22 | 0.43 |  | 0.16 | 0.33 |  | 0.11 | 0.07 |  | 0.58 | 1.83 |  | 10.67 | 28.78 |  | - | - |
| *Brevoortia tyrannus* | - | - |  | - | - |  | - | - |  | 0.19 | 0.19 |  | 0.67 | 2.30 |  | - | - |
| *Clupea harengus* | 4.84 | 10.52 |  | 0.49 | 0.47 |  | 0.63 | 1.06 |  | 2.14 | 2.43 |  | 5.33 | 10.43 |  | 3.85 | 3.34 |
| Clupeidae | 9.82 | 11.87 |  | 0.98 | 0.95 |  | 0.63 | 1.12 |  | 2.14 | 2.77 |  | 8.67 | 12.21 |  | 1.92 | 4.40 |
| Total Clupeiformes | *NA* | 25.04 |  | *NA* | 1.74 |  | *NA* | 2.79 |  | *NA* | 9.45 |  | *NA* | 53.73 |  | *NA* | 7.74 |

Table S3 continued

|  | *Merluccius bilinearis* | |  | *Urophycis chuss* | |  | *Urophycis tenuis* | |  | *Lophius americanus* | |  | *Squalus acanthias* | |  | *Gadus morhua* | |
| --- | --- | --- | --- | --- | --- | --- | --- | --- | --- | --- | --- | --- | --- | --- | --- | --- | --- |
| Prey taxon | %FO | %PM |  | %FO | %PM |  | %FO | %PM |  | %FO | %PM |  | %FO | %PM |  | %FO | %PM |
| **Chondrichthyes** |  |  |  |  |  |  |  |  |  |  |  |  |  |  |  |  |  |
| Rajidae | - | - |  | - | - |  | - | - |  | 0.19 | 0.02 |  | - | - |  | - | - |
| Chondrichthyes | - | - |  | - | - |  | - | - |  | - | - |  | 0.67 | 0.32 |  | - | - |
| Total Chondrichthyes | - | - |  | - | - |  | - | - |  | *NA* | 0.02 |  | *NA* | 0.32 |  | - | - |
|  |  |  |  |  |  |  |  |  |  |  |  |  |  |  |  |  |  |
| **Copepoda** |  |  |  |  |  |  |  |  |  |  |  |  |  |  |  |  |  |
| Copepoda | - | - |  | 0.33 | <0.01 |  | 0.11 | <0.01 |  | - | - |  | - | - |  | - | - |
|  |  |  |  |  |  |  |  |  |  |  |  |  |  |  |  |  |  |
| **Ctenophora** |  |  |  |  |  |  |  |  |  |  |  |  |  |  |  |  |  |
| Ctenophora | - | - |  | - | - |  | - | - |  | - | - |  | 1.33 | 0.16 |  | - | - |
|  |  |  |  |  |  |  |  |  |  |  |  |  |  |  |  |  |  |
| **Cumacea** |  |  |  |  |  |  |  |  |  |  |  |  |  |  |  |  |  |
| Cumacea | 0.07 | <0.01 |  | 0.16 | <0.01 |  | - | - |  | - | - |  | - | - |  | - | - |
|  |  |  |  |  |  |  |  |  |  |  |  |  |  |  |  |  |  |
| **Decapoda** |  |  |  |  |  |  |  |  |  |  |  |  |  |  |  |  |  |
| *Cancer* spp. | - | - |  | 2.45 | 1.45 |  | 0.63 | 0.30 |  | - | - |  | - | - |  | 13.46 | 17.65 |
| Crangonidae | 8.14 | 2.28 |  | 35.68 | 15.53 |  | 45.72 | 17.82 |  | 1.17 | 0.34 |  | 0.67 | <0.01 |  | 1.92 | 2.22 |
| Decapoda | 0.15 | <0.01 |  | 0.82 | 0.33 |  | 0.42 | 0.08 |  | - | - |  | - | - |  | - | - |
| Hippolytidae | 0.07 | 0.02 |  | 0.16 | 0.01 |  | 0.11 | 0.19 |  | - | - |  | - | - |  | 1.92 | 0.16 |
| *Homarus americanus* | - | - |  | 1.47 | 1.15 |  | 0.53 | 0.73 |  | - | - |  | - | - |  | 1.92 | 6.66 |
| Paguroidea | 0.07 | <0.01 |  | - | - |  | 0.11 | 0.01 |  | - | - |  | - | - |  | - | - |
| Pandalidae | 14.81 | 12.70 |  | 29.30 | 28.38 |  | 40.65 | 32.58 |  | 8.16 | 2.39 |  | 2.67 | 0.18 |  | 9.62 | 11.15 |
| *Pandalus borealis* | 1.17 | 1.65 |  | 2.29 | 4.13 |  | 2.64 | 4.08 |  | 4.47 | 2.40 |  | 0.67 | 0.35 |  | 3.85 | 2.10 |
| Pasiphaeidae | 0.22 | 0.05 |  | 0.16 | 0.13 |  | - | - |  | - | - |  | - | - |  | - | - |
| Thalassinidea | 0.07 | <0.01 |  | 1.96 | 2.40 |  | 1.27 | 1.22 |  | - | - |  | - | - |  | 1.92 | 2.22 |
| unidentified crab | 0.29 | <0.01 |  | 2.45 | 1.24 |  | 0.32 | 0.08 |  | 0.19 | <0.01 |  | - | - |  | 7.69 | 6.09 |
| unidentified shrimp | 6.60 | 1.86 |  | 11.78 | 5.27 |  | 11.83 | 4.54 |  | 1.17 | 0.15 |  | 1.33 | 0.12 |  | 15.38 | 9.69 |
| Total Decapoda | *NA* | 18.55 |  | *NA* | 60.01 |  | *NA* | 61.63 |  | *NA* | 5.28 |  | *NA* | 0.66 |  | *NA* | 57.93 |

Table S3 continued

|  | *Merluccius bilinearis* | |  | *Urophycis chuss* | |  | *Urophycis tenuis* | |  | *Lophius americanus* | |  | *Squalus acanthias* | |  | *Gadus morhua* | |
| --- | --- | --- | --- | --- | --- | --- | --- | --- | --- | --- | --- | --- | --- | --- | --- | --- | --- |
| Prey taxon | %FO | %PM |  | %FO | %PM |  | %FO | %PM |  | %FO | %PM |  | %FO | %PM |  | %FO | %PM |
| **Echinodermata** |  |  |  |  |  |  |  |  |  |  |  |  |  |  |  |  |  |
| Ophiuroidea | 0.15 | <0.01 |  | 2.29 | 0.53 |  | 0.74 | <0.01 |  | - | - |  | - | - |  | - | - |
|  |  |  |  |  |  |  |  |  |  |  |  |  |  |  |  |  |  |
| **Euphausiacea** |  |  |  |  |  |  |  |  |  |  |  |  |  |  |  |  |  |
| Euphausiidae | 15.91 | 8.49 |  | 12.60 | 6.72 |  | 7.60 | 1.87 |  | 0.97 | 0.48 |  | 4.67 | 1.43 |  | 3.85 | 0.52 |
|  |  |  |  |  |  |  |  |  |  |  |  |  |  |  |  |  |  |
| **Gadiformes** |  |  |  |  |  |  |  |  |  |  |  |  |  |  |  |  |  |
| *Enchelyopus cimbrius* | 0.07 | 0.13 |  | - | - |  | 0.42 | 1.27 |  | 9.13 | 11.83 |  | - | - |  | - | - |
| Gadiformes | 1.32 | 1.16 |  | 0.65 | 0.72 |  | 1.90 | 1.33 |  | 4.27 | 5.61 |  | - | - |  | - | - |
| *Gadus morhua* | - | - |  | - | - |  | - | - |  | 0.19 | 0.21 |  | - | - |  | - | - |
| *Melanogrammus aeglefinus* | - | - |  | - | - |  | - | - |  | 0.78 | 1.03 |  | - | - |  | - | - |
| *Merluccius bilinearis* | 20.38 | 31.28 |  | 4.09 | 4.60 |  | 9.50 | 14.16 |  | 13.59 | 14.93 |  | 1.33 | 1.65 |  | 11.54 | 10.36 |
| *Urophycis chuss* | 0.07 | 0.08 |  | 0.16 | 0.17 |  | 0.32 | 0.50 |  | 2.33 | 3.15 |  | 0.67 | 0.51 |  | - | - |
| *Urophycis* spp. | 0.73 | 0.81 |  | 1.31 | 0.63 |  | 1.16 | 0.74 |  | 3.11 | 3.89 |  | - | - |  | - | - |
| *Urophycis tenuis* | 0.37 | 0.84 |  | - | - |  | - | - |  | 0.78 | 0.54 |  | - | - |  | - | - |
| Total Gadiformes | *NA* | 34.31 |  | *NA* | 6.11 |  | *NA* | 18.01 |  | *NA* | 41.19 |  | *NA* | 2.17 |  | *NA* | 10.36 |
|  |  |  |  |  |  |  |  |  |  |  |  |  |  |  |  |  |  |
| **Hydrozoa** |  |  |  |  |  |  |  |  |  |  |  |  |  |  |  |  |  |
| Hydrozoa | - | - |  | 0.16 | <0.01 |  | - | - |  | - | - |  | - | - |  | - | - |
|  |  |  |  |  |  |  |  |  |  |  |  |  |  |  |  |  |  |
| **Isopoda** |  |  |  |  |  |  |  |  |  |  |  |  |  |  |  |  |  |
| Isopoda | 0.15 | <0.01 |  | 0.16 | 0.03 |  | - | - |  | - | - |  | - | - |  | - | - |
|  |  |  |  |  |  |  |  |  |  |  |  |  |  |  |  |  |  |
| **Labriformes** |  |  |  |  |  |  |  |  |  |  |  |  |  |  |  |  |  |
| *Tautogolabrus adspersus* | - | - |  | - | - |  | - | - |  | 0.19 | 0.13 |  | - | - |  | - | - |
|  |  |  |  |  |  |  |  |  |  |  |  |  |  |  |  |  |  |
| **Lophiiformes** |  |  |  |  |  |  |  |  |  |  |  |  |  |  |  |  |  |
| *Lophius americanus* | - | - |  | - | - |  | 0.11 | 0.33 |  | 0.58 | 0.66 |  | - | - |  | - | - |
|  |  |  |  |  |  |  |  |  |  |  |  |  |  |  |  |  |  |
| **Mollusca** |  |  |  |  |  |  |  |  |  |  |  |  |  |  |  |  |  |
| Bivalvia | 0.07 | <0.01 |  | 2.78 | 0.07 |  | 1.37 | <0.01 |  | 0.39 | <0.01 |  | - | - |  | 1.92 | 0.02 |

Table S3 continued

|  | *Merluccius bilinearis* | |  | *Urophycis chuss* | |  | *Urophycis tenuis* | |  | *Lophius americanus* | |  | *Squalus acanthias* | |  | *Gadus morhua* | |
| --- | --- | --- | --- | --- | --- | --- | --- | --- | --- | --- | --- | --- | --- | --- | --- | --- | --- |
| Prey taxon | %FO | %PM |  | %FO | %PM |  | %FO | %PM |  | %FO | %PM |  | %FO | %PM |  | %FO | %PM |
| Mollusca | - | - |  | 0.49 | 0.10 |  | - | - |  | 0.19 | 0.02 |  | - | - |  | - | - |
| Total Mollusca | *NA* | <0.01 |  | *NA* | 0.17 |  | *NA* | <0.01 |  | *NA* | 0.02 |  | - | - |  | *NA* | 0.02 |
|  |  |  |  |  |  |  |  |  |  |  |  |  |  |  |  |  |  |
| **Mysida** |  |  |  |  |  |  |  |  |  |  |  |  |  |  |  |  |  |
| Mysida | 3.52 | 0.61 |  | 8.18 | 2.41 |  | 6.55 | 1.89 |  | 0.39 | 0.01 |  | 0.67 | <0.01 |  | 3.85 | 0.26 |
|  |  |  |  |  |  |  |  |  |  |  |  |  |  |  |  |  |  |
| **Ophidiiformes** |  |  |  |  |  |  |  |  |  |  |  |  |  |  |  |  |  |
| *Ophidion barbatum* | - | - |  | - | - |  | - | - |  | 1.94 | 2.05 |  | - | - |  | - | - |
|  |  |  |  |  |  |  |  |  |  |  |  |  |  |  |  |  |  |
| **Pleuronectiformes** |  |  |  |  |  |  |  |  |  |  |  |  |  |  |  |  |  |
| Bothidae | - | - |  | - | - |  | - | - |  | - | - |  | 0.67 | 0.16 |  | - | - |
| *Glyptocephalus cynoglossus* | - | - |  | - | - |  | - | - |  | 0.58 | 0.65 |  | - | - |  | - | - |
| *Hippoglossoides platessoides* | 0.15 | 0.16 |  | 0.49 | 1.04 |  | 0.32 | 0.15 |  | 2.52 | 2.22 |  | - | - |  | - | - |
| Pleuronectiformes | 0.07 | 0.07 |  | 0.16 | <0.01 |  | 0.74 | 0.21 |  | 1.75 | 1.44 |  | 0.67 | 0.26 |  | - | - |
| *Pseudopleuronectes americanus* | - | - |  | - | - |  | - | - |  | 0.58 | 1.56 |  | - | - |  | - | - |
| Total Pleuronectiformes | *NA* | 0.23 |  | *NA* | 1.04 |  | *NA* | 0.36 |  | *NA* | 5.87 |  | *NA* | 0.42 |  | - | - |
|  |  |  |  |  |  |  |  |  |  |  |  |  |  |  |  |  |  |
| **Scombriformes** |  |  |  |  |  |  |  |  |  |  |  |  |  |  |  |  |  |
| *Peprilus triacanthus* | 1.03 | 2.16 |  | 0.16 | 0.30 |  | 0.53 | 0.38 |  | 0.39 | 0.58 |  | 1.33 | 0.71 |  | - | - |
| *Scomber scombrus* | 0.15 | 0.62 |  | 0.16 | 0.50 |  | 0.11 | 0.19 |  | 0.58 | 1.52 |  | 3.33 | 6.38 |  | - | - |
| Total Scombriformes | *NA* | 2.78 |  | *NA* | 0.79 |  | *NA* | 0.56 |  | *NA* | 2.10 |  | *NA* | 7.09 |  | - | - |
|  |  |  |  |  |  |  |  |  |  |  |  |  |  |  |  |  |  |
| **Scorpaeniformes** |  |  |  |  |  |  |  |  |  |  |  |  |  |  |  |  |  |
| *Cryptacanthodes maculatus* | - | - |  | 0.16 | 0.61 |  | 1.37 | 3.02 |  | 1.17 | 1.47 |  | 2.00 | 3.04 |  | - | - |
| *Cyclopterus* spp. | - | - |  | - | - |  | - | - |  | 0.19 | 0.12 |  | - | - |  | - | - |
| *Hemitripterus americanus* | - | - |  | - | - |  | - | - |  | 0.19 | 0.41 |  | - | - |  | - | - |
| *Lycenchelys verrilli* | - | - |  | - | - |  | 0.32 | 0.23 |  | - | - |  | 0.67 | 0.04 |  | - | - |
| *Pholis gunnellus* | - | - |  | - | - |  | - | - |  | 0.19 | 0.62 |  | - | - |  | - | - |
| *Sebastes fasciatus* | 0.81 | 0.72 |  | 1.96 | 1.38 |  | 2.75 | 0.75 |  | 1.17 | 0.28 |  | - | - |  | - | - |

Table S3 continued

|  | *Merluccius bilinearis* | |  | *Urophycis chuss* | |  | *Urophycis tenuis* | |  | *Lophius americanus* | |  | *Squalus acanthias* | |  | *Gadus morhua* | |
| --- | --- | --- | --- | --- | --- | --- | --- | --- | --- | --- | --- | --- | --- | --- | --- | --- | --- |
| Prey taxon | %FO | %PM |  | %FO | %PM |  | %FO | %PM |  | %FO | %PM |  | %FO | %PM |  | %FO | %PM |
| Stichaeidae | - | - |  | - | - |  | 0.11 | 0.04 |  | 0.19 | 0.21 |  | - | - |  | - | - |
| Zoarcidae | - | - |  | - | - |  | 0.11 | 0.03 |  | - | - |  | - | - |  | - | - |
| Total Scorpaeniformes | *NA* | 0.72 |  | *NA* | 1.99 |  | *NA* | 4.08 |  | *NA* | 3.11 |  | *NA* | 3.08 |  | *NA* | 0.00 |
|  |  |  |  |  |  |  |  |  |  |  |  |  |  |  |  |  |  |
| **Trachiniformes** |  |  |  |  |  |  |  |  |  |  |  |  |  |  |  |  |  |
| *Amodytes* spp. | 0.07 | 0.23 |  | - | - |  | - | - |  | 0.19 | 0.03 |  | - | - |  | - | - |
|  |  |  |  |  |  |  |  |  |  |  |  |  |  |  |  |  |  |
| **Unidentifed contents** |  |  |  |  |  |  |  |  |  |  |  |  |  |  |  |  |  |
| unidentified Invertebrata | 2.57 | 0.43 |  | 9.00 | 3.51 |  | 3.48 | 0.76 |  | 0.97 | 0.57 |  | 7.33 | 3.39 |  | 13.46 | 7.24 |
| unidentified Teleostei | 9.09 | 7.10 |  | 9.17 | 5.51 |  | 9.19 | 4.50 |  | 31.46 | 25.97 |  | 12.67 | 10.07 |  | 15.38 | 9.83 |
| unidentified remains | 0.88 | <0.01 |  | 1.80 | 0.88 |  | 0.63 | 0.01 |  | 0.78 | 0.13 |  | 2.00 | 0.19 |  | 5.77 | 2.24 |
| Total unidentified contents | *NA* | 7.53 |  | *NA* | 9.90 |  | *NA* | 5.27 |  | *NA* | 26.67 |  | *NA* | 13.66 |  | *NA* | 19.30 |
|  |  |  |  |  |  |  |  |  |  |  |  |  |  |  |  |  |  |
| **Empty (no contents)** |  |  |  |  |  |  |  |  |  |  |  |  |  |  |  |  |  |
| Total empty stomachs | 19.21 | *NA* |  | 14.73 | *NA* |  | 5.49 | *NA* |  | 25.43 | *NA* |  | 35.33 | *NA* |  | 15.38 | *NA* |

Figure S2. Scatter plots depicting relationships between tow depth and the proportional mass of taxa in spring tow catches. Results of Spearman rank correlation are displayed in each plot. Blue points represent individual tows. Red lines show the fit of locally estimated scatterplot smoothing (LOESS). Statistical significance of a non-monotonic relationship should be considered a spurious correlation.


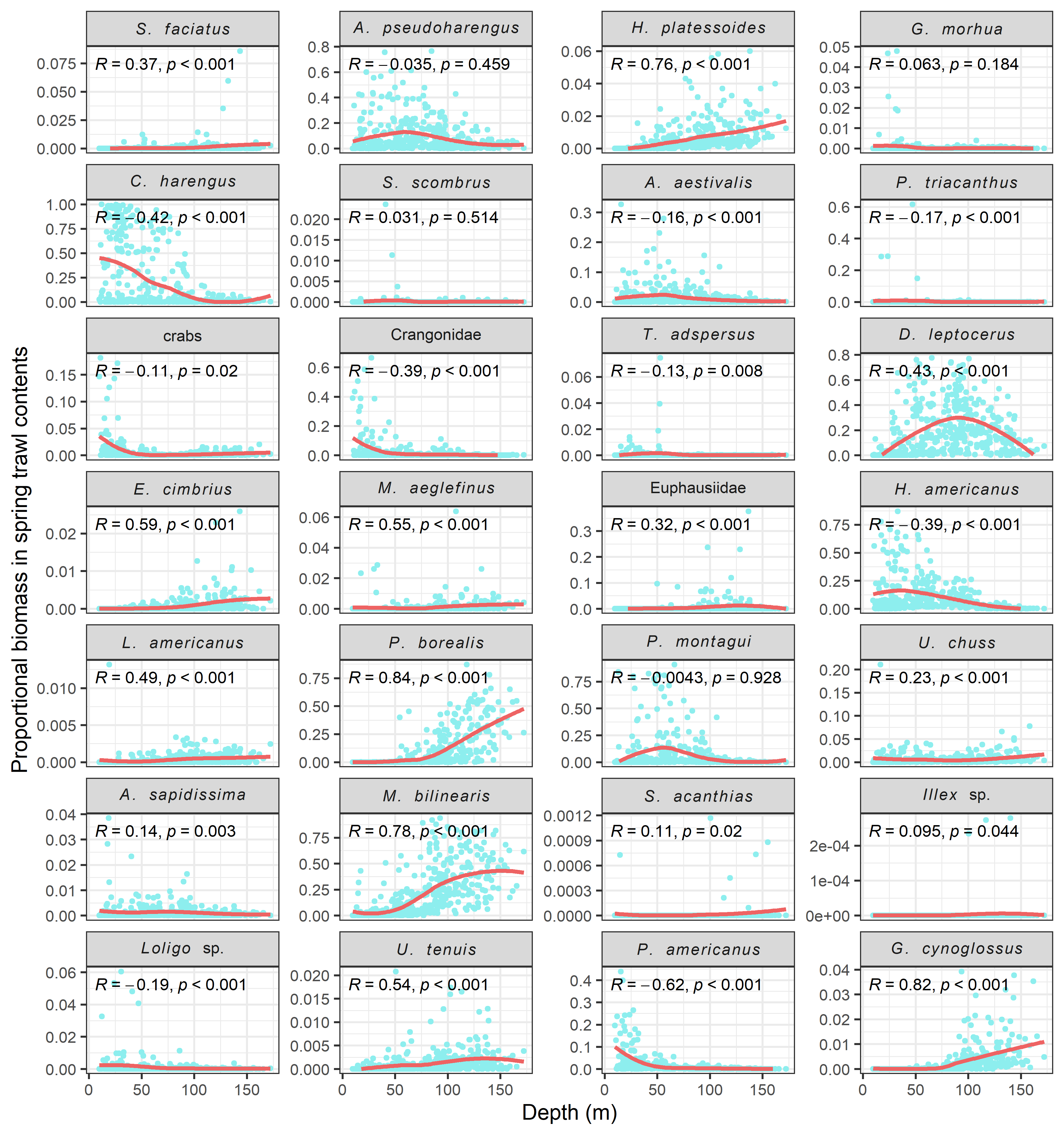


Figure S3. Scatter plots depicting relationships between tow depth and the proportional mass of taxa in fall tow catches. Results of Spearman rank correlation are displayed in each plot. Blue points represent individual tows. Red lines show the fit of locally estimated scatterplot smoothing (LOESS). Statistical significance of a non-monotonic relationship should be considered a spurious correlation.


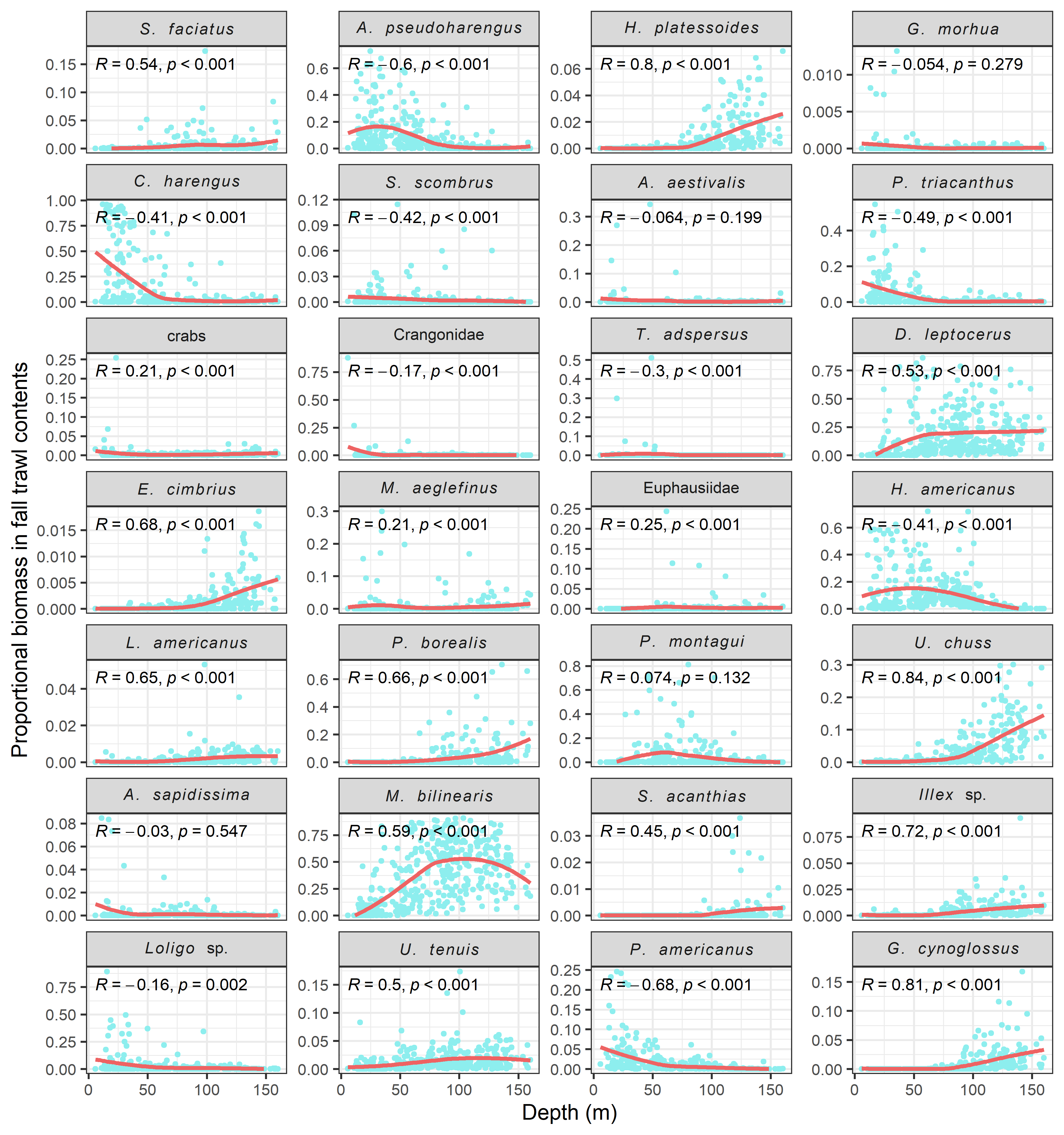


Figure S4. Scatter plots depicting relationships between survey year and the proportional mass of taxa in spring tow catches. Results of Spearman rank correlation are displayed in each plot. Blue points represent individual tows. Red lines show the fit of locally estimated scatterplot smoothing (LOESS). Statistical significance of a non-monotonic relationship should be considered a spurious correlation.


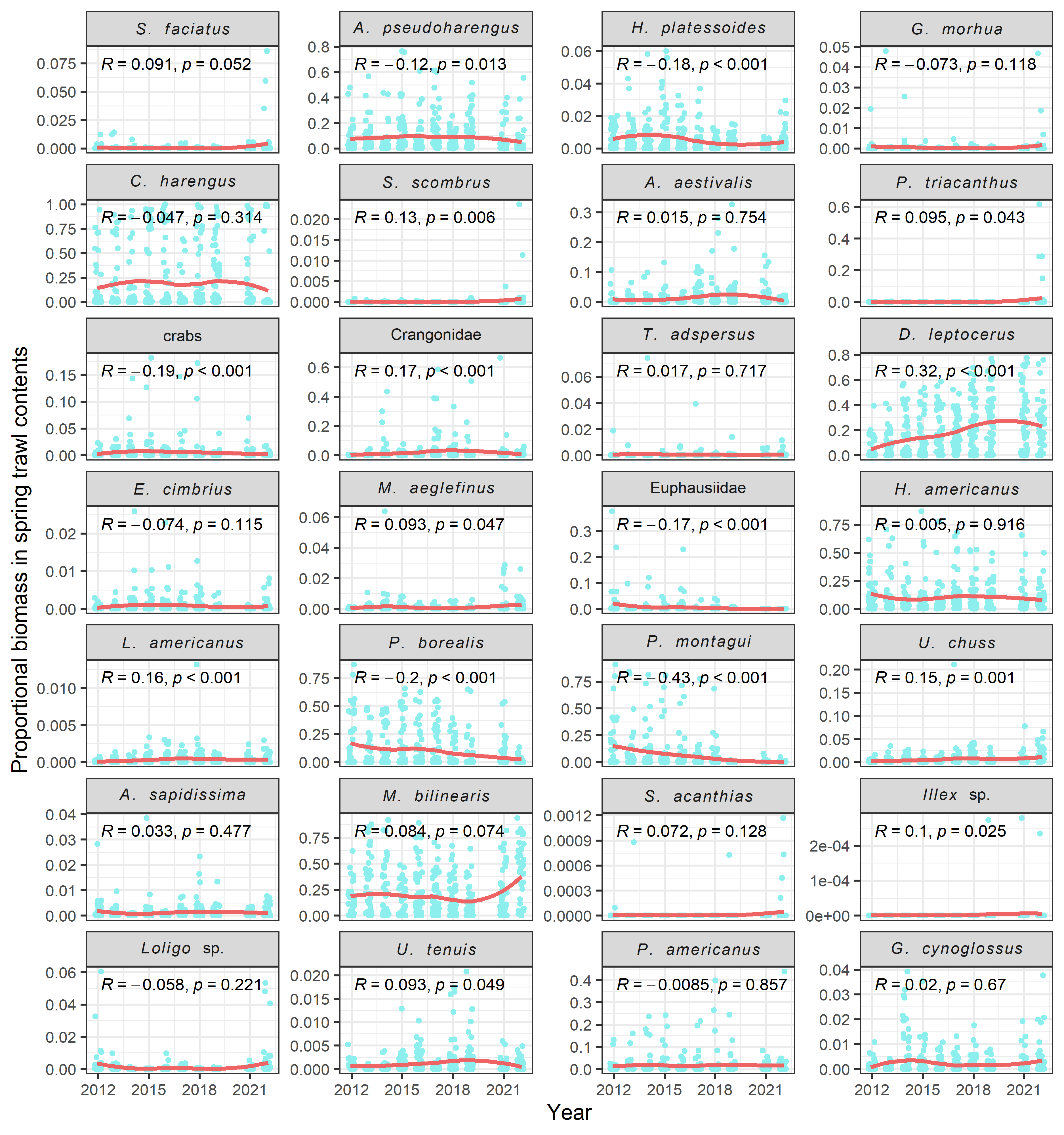


Figure S5. Scatter plots depicting relationships between survey year and the proportional mass of taxa in fall tow catches. Results of Spearman rank correlation are displayed in each plot. Blue points represent individual tows. Red lines show the fit of locally estimated scatterplot smoothing (LOESS). Statistical significance of a non-monotonic relationship should be considered a spurious correlation.


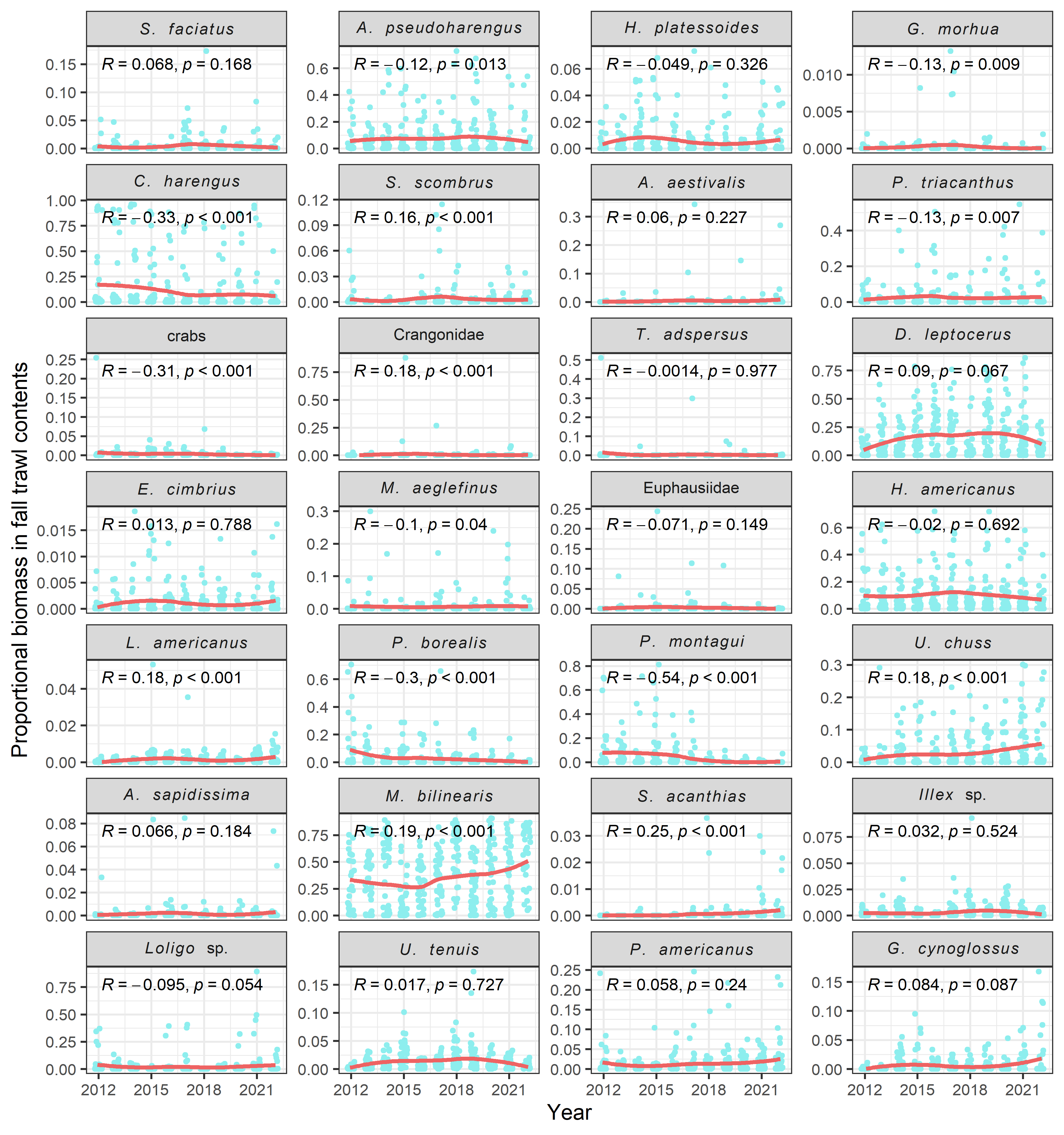


Figure S6. Scatter plots depicting relationships between longitudinal position of tow and the proportional mass of taxa in spring tow catches. Results of Spearman rank correlation are displayed in each plot. Blue points represent individual tows. Red lines show the fit of locally estimated scatterplot smoothing (LOESS). Statistical significance of a non-monotonic relationship should be considered a spurious correlation.


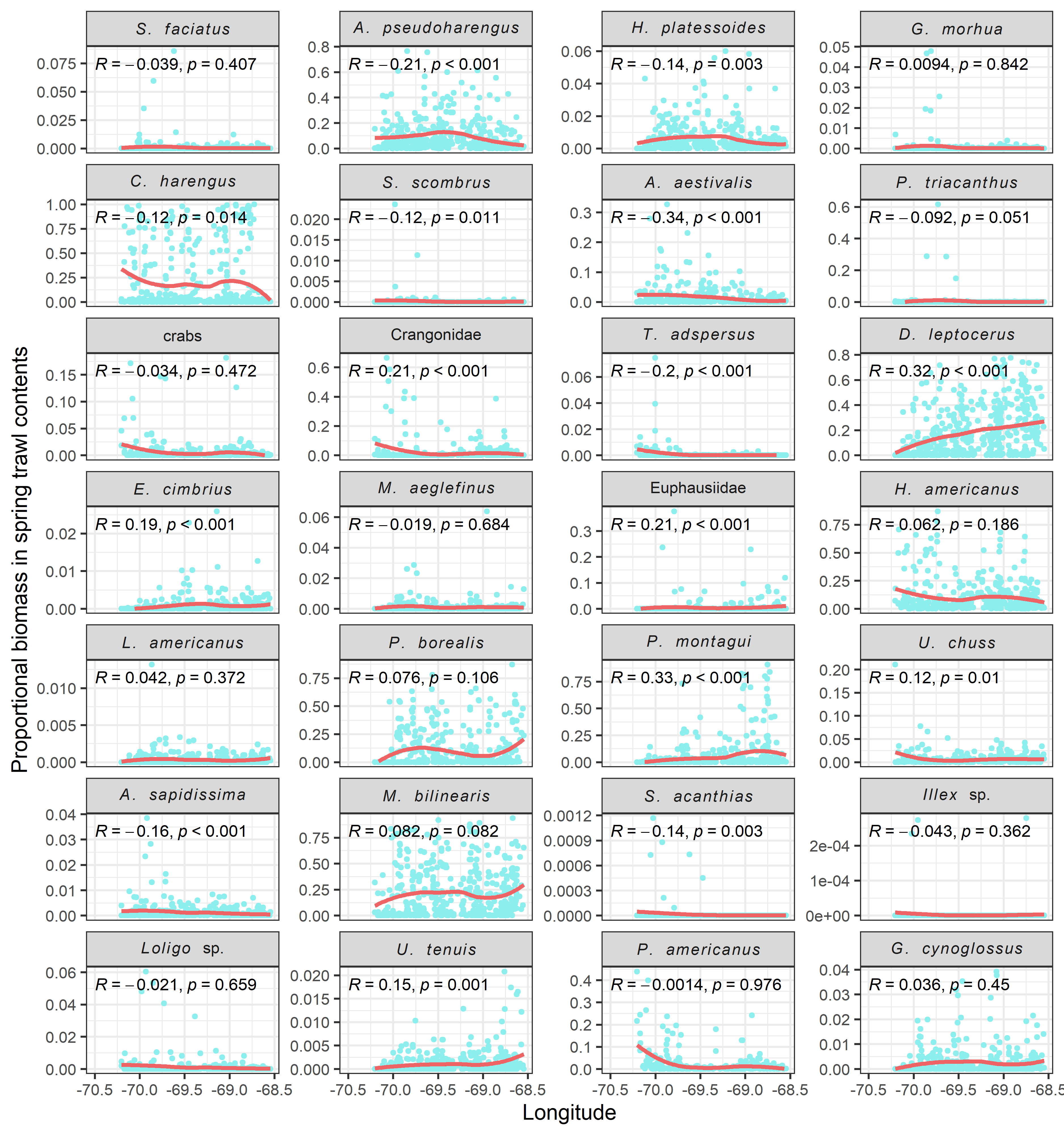


Figure S7. Scatter plots depicting relationships between longitudinal position of tow and the proportional mass of taxa in fall tow catches. Results of Spearman rank correlation are displayed in each plot. Blue points represent individual tows. Red lines show the fit of locally estimated scatterplot smoothing (LOESS). Statistical significance of a non-monotonic relationship should be considered a spurious correlation.


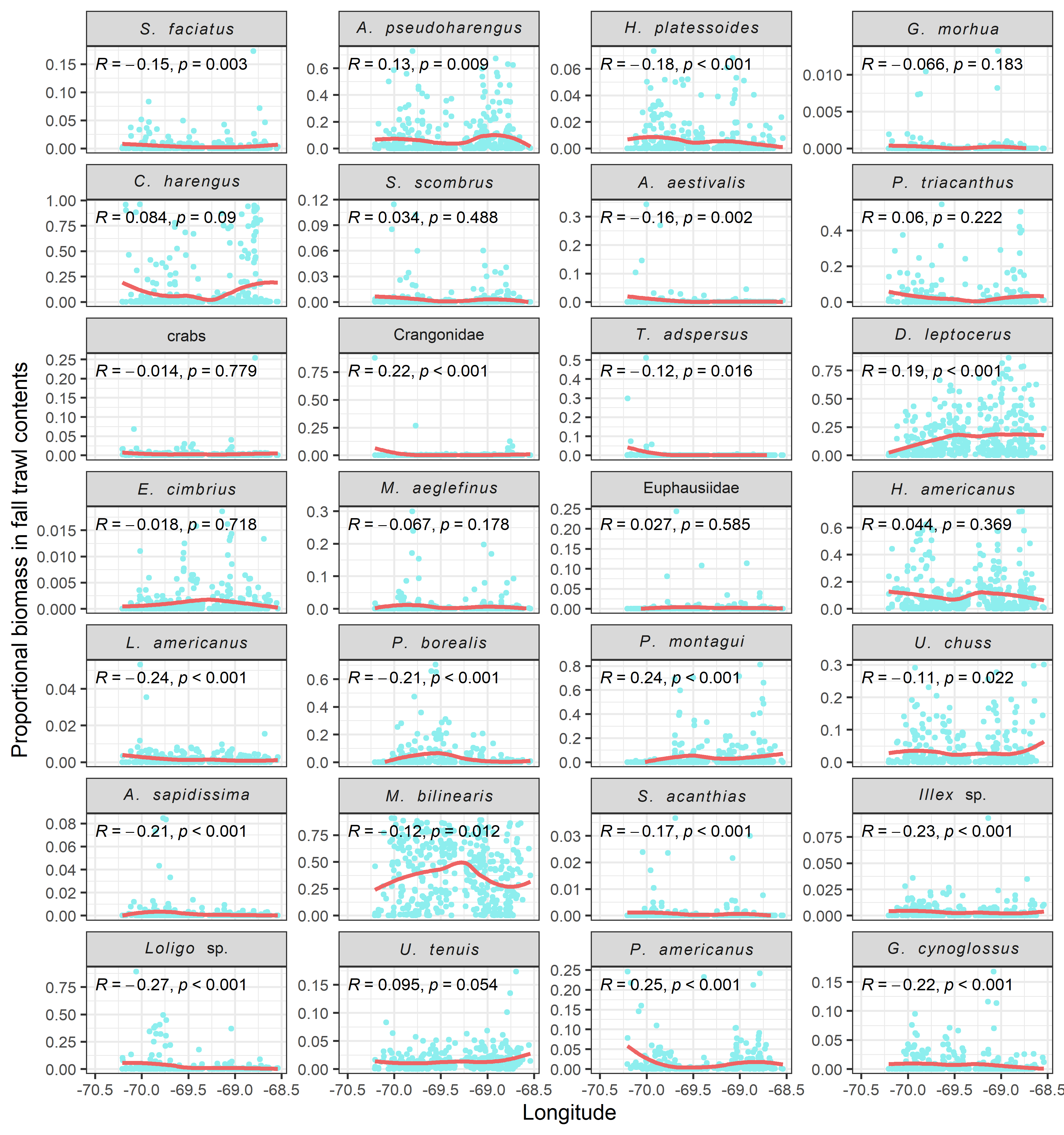


Figure S8. Scatter plots depicting relationships between bottom temperature and the proportional mass of taxa in spring tow catches. Results of Spearman rank correlation are displayed in each plot. Blue points represent individual tows. Red lines show the fit of locally estimated scatterplot smoothing (LOESS). Statistical significance of a non-monotonic relationship should be considered a spurious correlation.


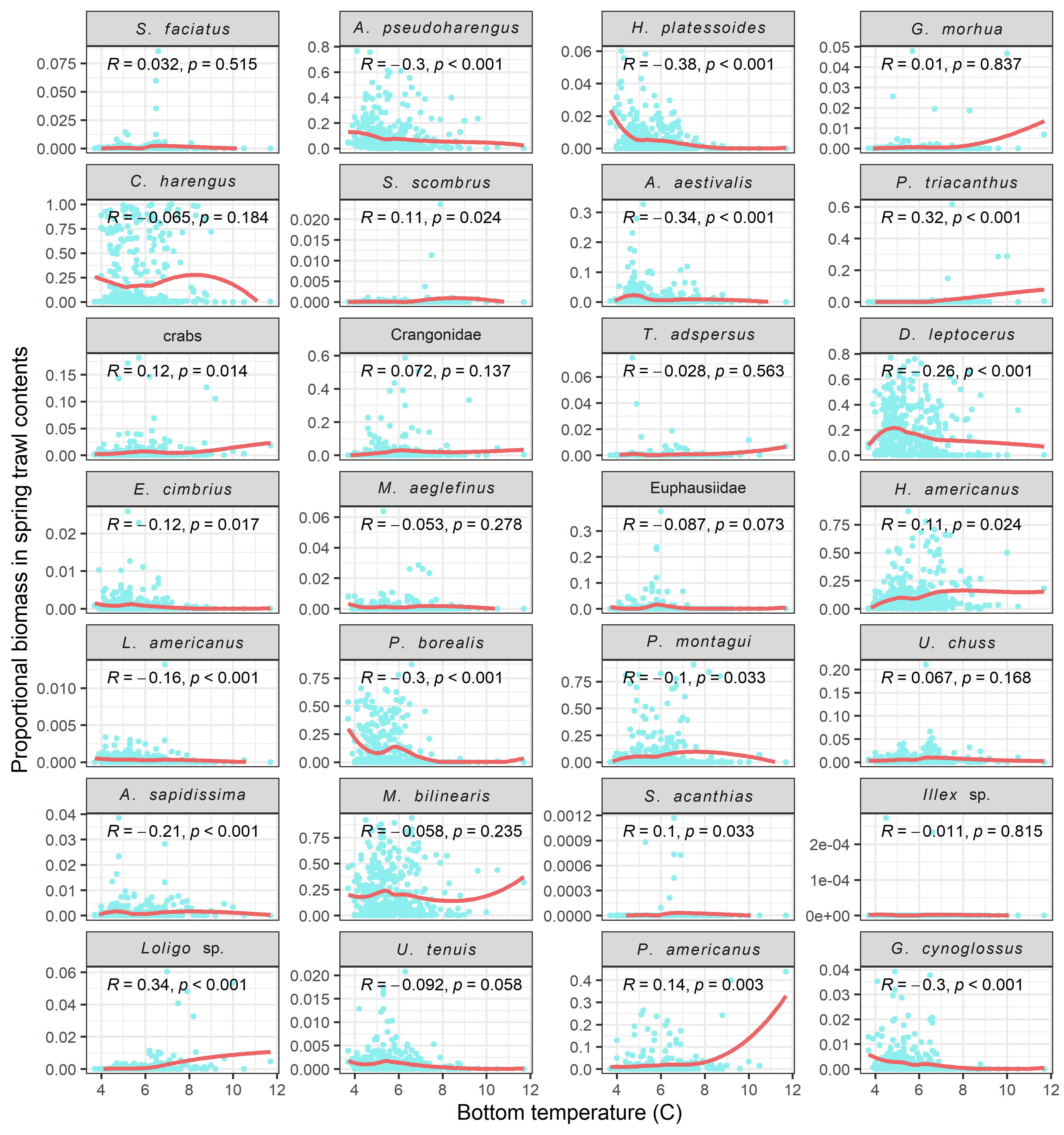


Figure S9. Scatter plots depicting relationships between bottom temperature and the proportional mass of taxa in fall tow catches. Results of Spearman rank correlation are displayed in each plot. Blue points represent individual tows. Red lines show the fit of locally estimated scatterplot smoothing (LOESS). Statistical significance of a non-monotonic relationship should be considered a spurious correlation.


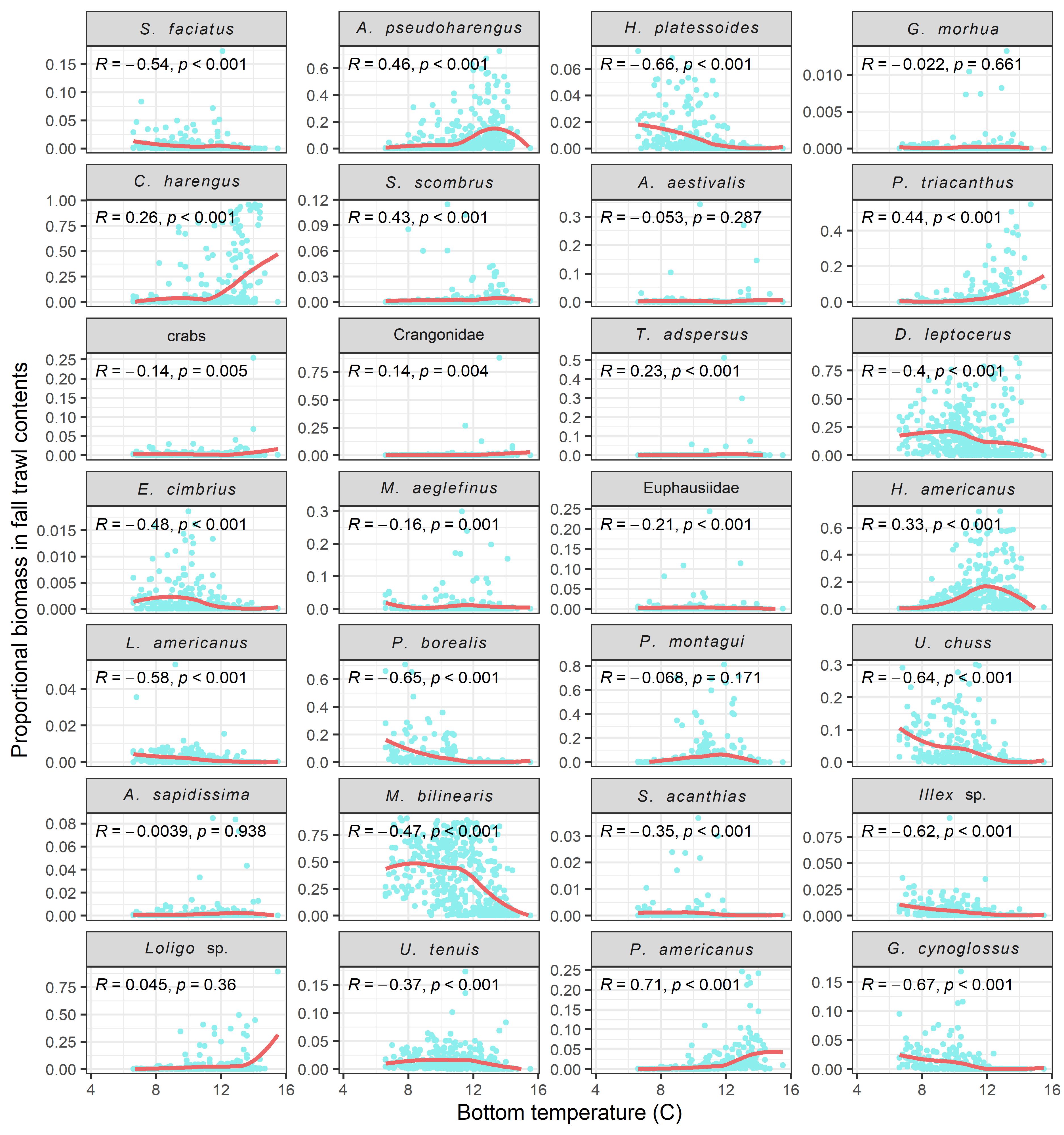


Figure S10. Scatter plots and simple linear regression of body lengths of the six focal predators (*Gadus morhua*, *Lophius americanus, Merluccius bilinearis*, *Squalus acanthias*, *Urophycis chuss*, and *Urophycis tenuis*) by tow depth in spring and fall. Each point represents an observed length. For each species-season combination, simple linear regression was used to determine significance of the slope. Red lines depict the median linear regression fit for statistically significant relationships (p < 0.05).


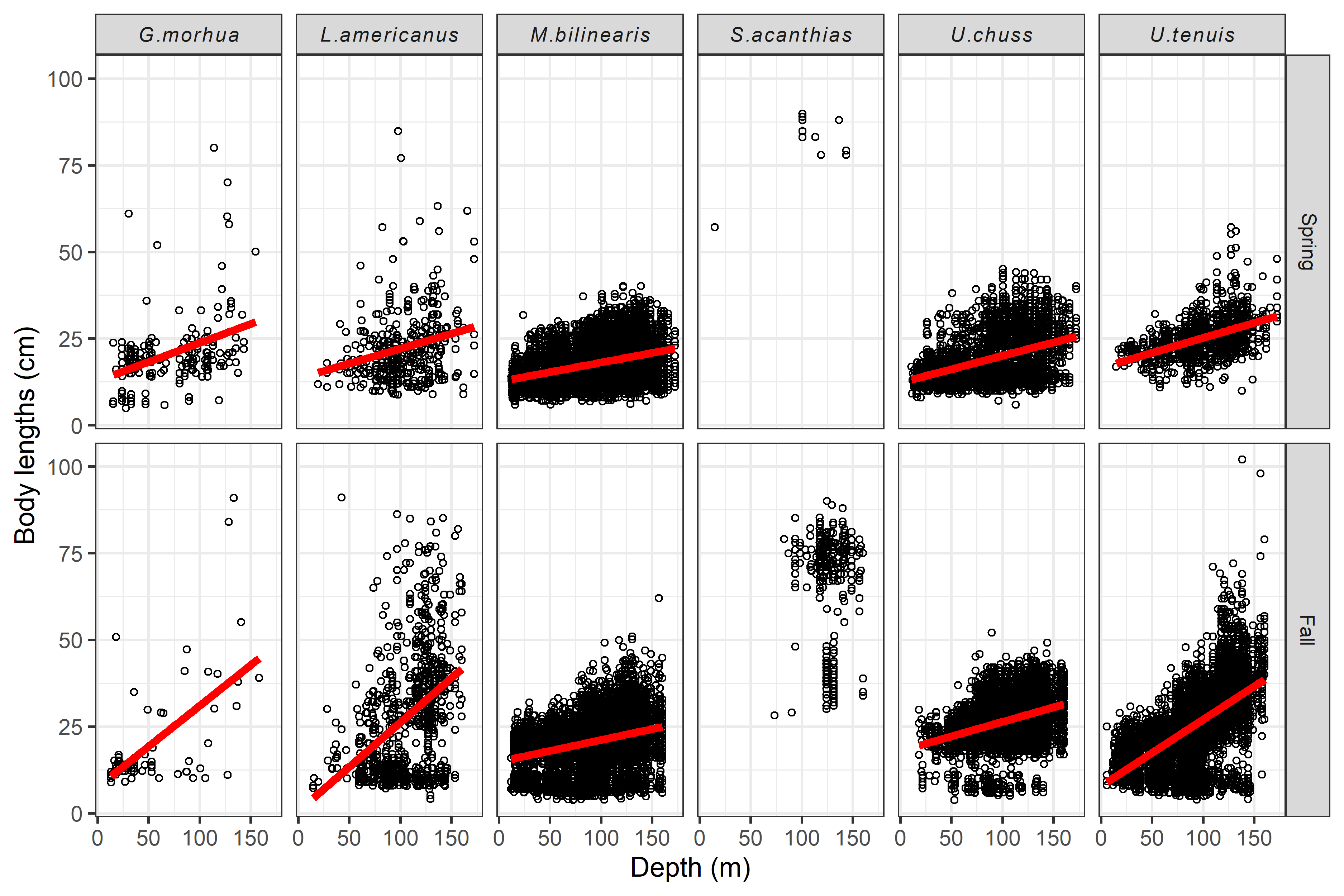


Figure S11. Scatter plots and simple linear regression of body lengths of five piscine prey (*Alosa aestivalis*, *Alosa pseudoharengus*, *Alosa sapidissima*, *Clupea harengus*, and *Peprilus triacanthus*) by tow depth in spring and fall. Each point represents an observed length. For each species-season combination, simple linear regression was used to determine significance of the slope. Red lines depict the median linear regression fit for statistically significant relationships (p < 0.05).


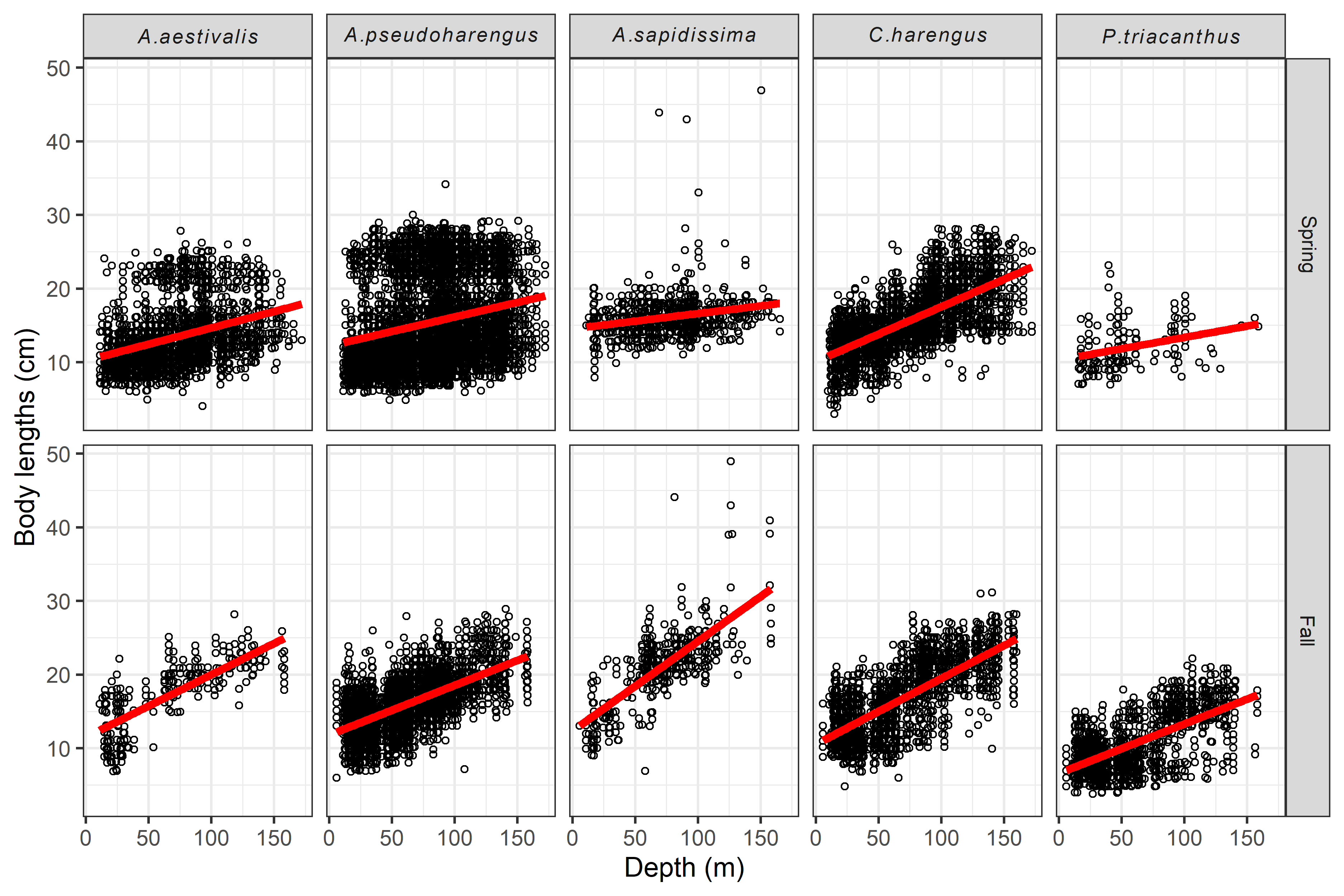


Figure S12. Cumulative Shannon-Wiener diversity in stomach contents of *Merluccius bilinearis*, *Urophycis chuss*, *Urophycis tenuis*, *Lophius americanus*, *Squalus acanthias*, and *Gadus morhua* as a function of randomized stomach number (up to 150 stomachs and excluding empty stomachs) over both sampling seasons combined (A) and in spring (B) and fall (C) separately. Curves that do not asymptote horizontally indicate that sample sizes are insufficient to fully characterize diets at that level.


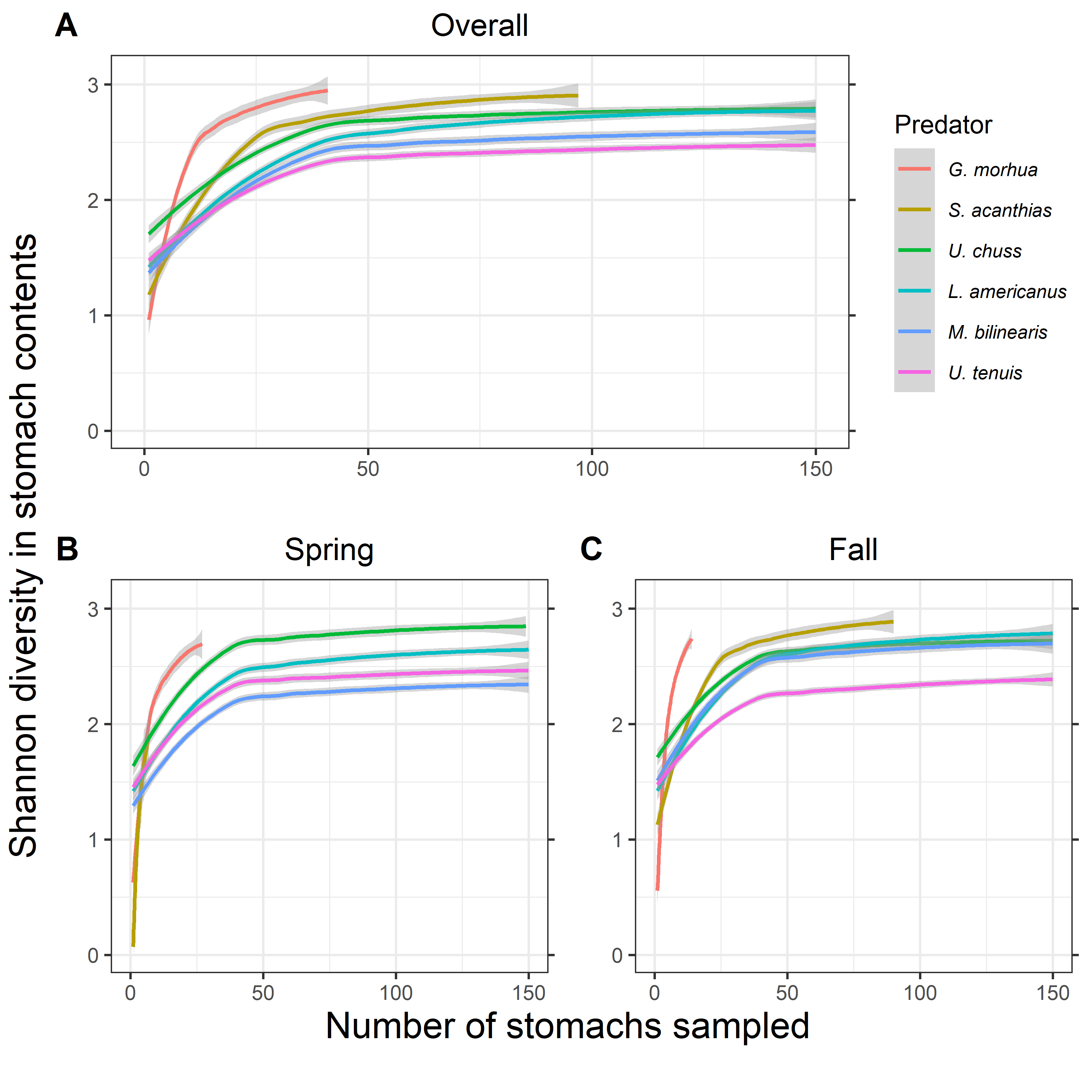


Table S4. Mean and standard deviation of tow-level proportional mass by prey taxon in stomach contents of *Merluccius bilinearis*, *Urophycis chuss*, *Urophycis tenuis*, and *Lophius americanus* sampled in nearshore Gulf of Maine, and results of permutational one-way ANOVA comparing the proportional masses between seasons. Only prey taxa with significant differences between seasons are included in this table. Sample sizes were not sufficient to assess seasonal variation in the other two focal predator species (*Squalas acanthias* and *Gadus morhua*).

| Predator | Prey taxon | x̄_SPRING_ | SD_SPRING_ | x̄_Fall_ | SD_Fall_ | Z | P.value |
| --- | --- | --- | --- | --- | --- | --- | --- |
| *M. bilinearis* |  |  |  |  |  |  |  |
|  | Clupeidae | 0.083 | 0.161 | 0.187 | 0.327 | -2.403 | 0.016 |
|  | Crangonidae | 0.006 | 0.020 | 0.047 | 0.161 | -2.173 | 0.030 |
|  | Euphausiidae | 0.104 | 0.201 | 0.031 | 0.149 | 2.356 | 0.018 |
|  | *M. bilinearis* | 0.476 | 0.363 | 0.106 | 0.242 | 6.068 | <.001 |
|  | Pandalidae | 0.082 | 0.189 | 0.165 | 0.302 | -1.977 | 0.048 |
|  | *Peprilus triacanthus* | <.001 | <.001 | 0.051 | 0.159 | -2.708 | 0.007 |
|  | Teleostei | 0.031 | 0.121 | 0.163 | 0.292 | -3.469 | 0.001 |
| *U. chuss* |  |  |  |  |  |  |  |
|  | Crangonidae | 0.009 | 0.021 | 0.131 | 0.188 | -2.055 | 0.040 |
|  | Euphausiidae | 0.216 | 0.361 | 0.020 | 0.061 | 2.835 | 0.005 |
| *U. tenuis* |  |  |  |  |  |  |  |
|  | Pleocyemata | 0.057 | 0.114 | 0.019 | 0.052 | 2.011 | 0.044 |
|  | Euphausiidae | 0.072 | 0.196 | 0.002 | 0.008 | 2.576 | 0.010 |
|  | *M. bilinearis* | 0.342 | 0.329 | 0.108 | 0.212 | 3.537 | <.001 |
|  | Mysida | 0.013 | 0.044 | <.001 | 0.001 | 2.074 | 0.038 |
|  | Pandalidae | 0.225 | 0.237 | 0.387 | 0.284 | -2.411 | 0.016 |
| *L. americanus* |  |  |  |  |  |  |  |
|  | Crustacea | 0.049 | 0.110 | <.001 | <.001 | 2.144 | 0.032 |
|  | *Enchelyopus cimbrius* | 0.231 | 0.367 | 0.042 | 0.112 | 2.195 | 0.028 |
|  | Pandalidae | 0.017 | 0.025 | 0.004 | 0.011 | 1.999 | 0.046 |

Figure S13. Scatter plots depicting relationships between tow-level proportional mass of prey in stomach contents of *Merluccius bilinearis* and tow depth, bottom temperature, longitude and survey year. Results of Spearman rank correlation are displayed in each plot, and only prey taxa with a p-value <0.05 are included. Blue points represent individual tow-level values. Red lines show the fit of locally estimated scatterplot smoothing (LOESS). Statistical significance of a non-monotonic relationship should be considered a spurious correlation.


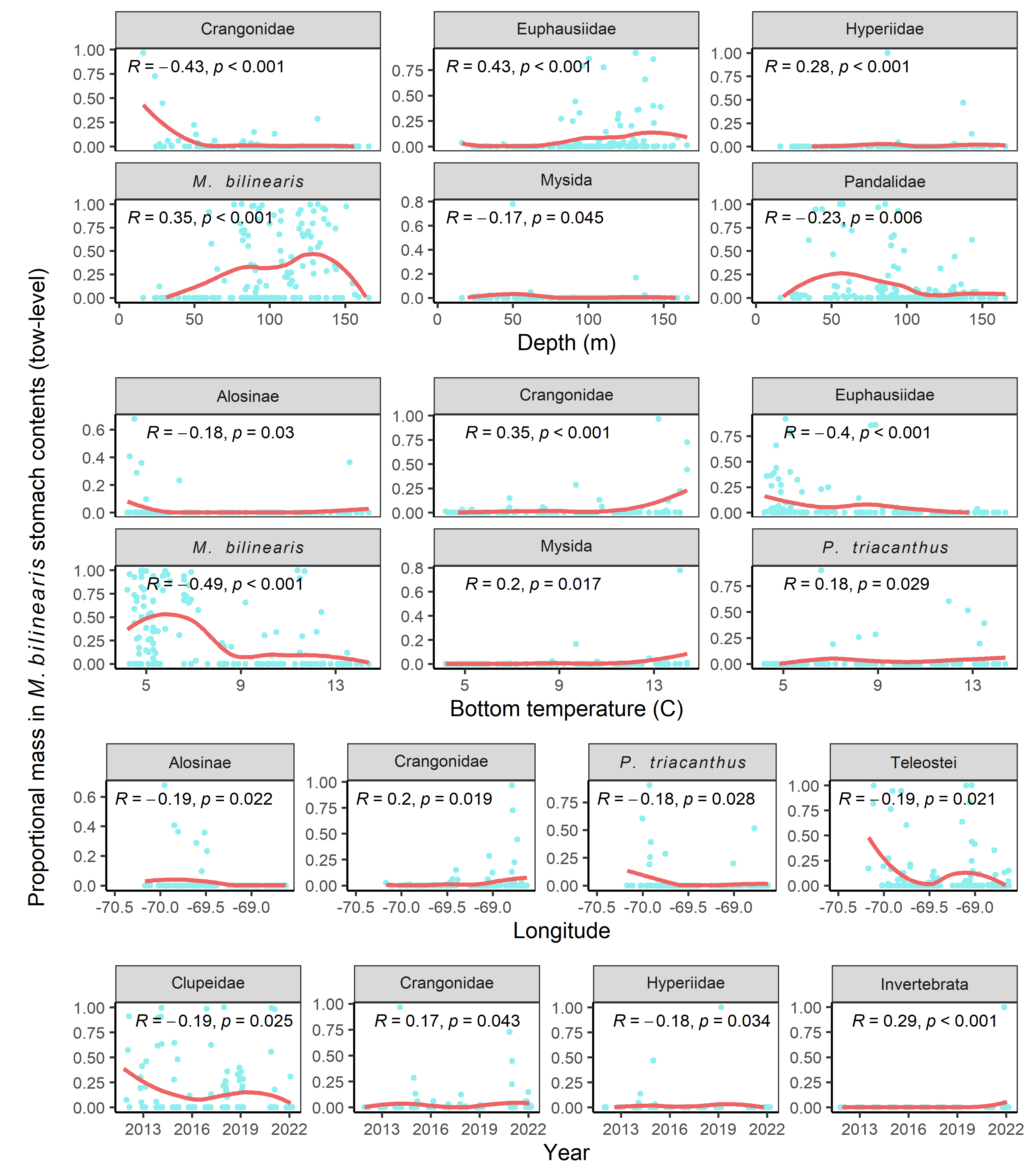


Figure S14. Scatter plots depicting relationships between tow-level proportional mass of prey in stomach contents of *Urophycis chuss* and tow depth, bottom temperature, longitude and survey year. Results of Spearman rank correlation are displayed in each plot, and only prey taxa with a p-value <0.05 are included. However, because only one prey taxon had a significant correlation with year, the taxon with the next lowest p-value (Clupeidae) is also shown. Blue points represent individual tow-level values. Red lines show the fit of locally estimated scatterplot smoothing (LOESS). Statistical significance of a non-monotonic relationship should be considered a spurious correlation.


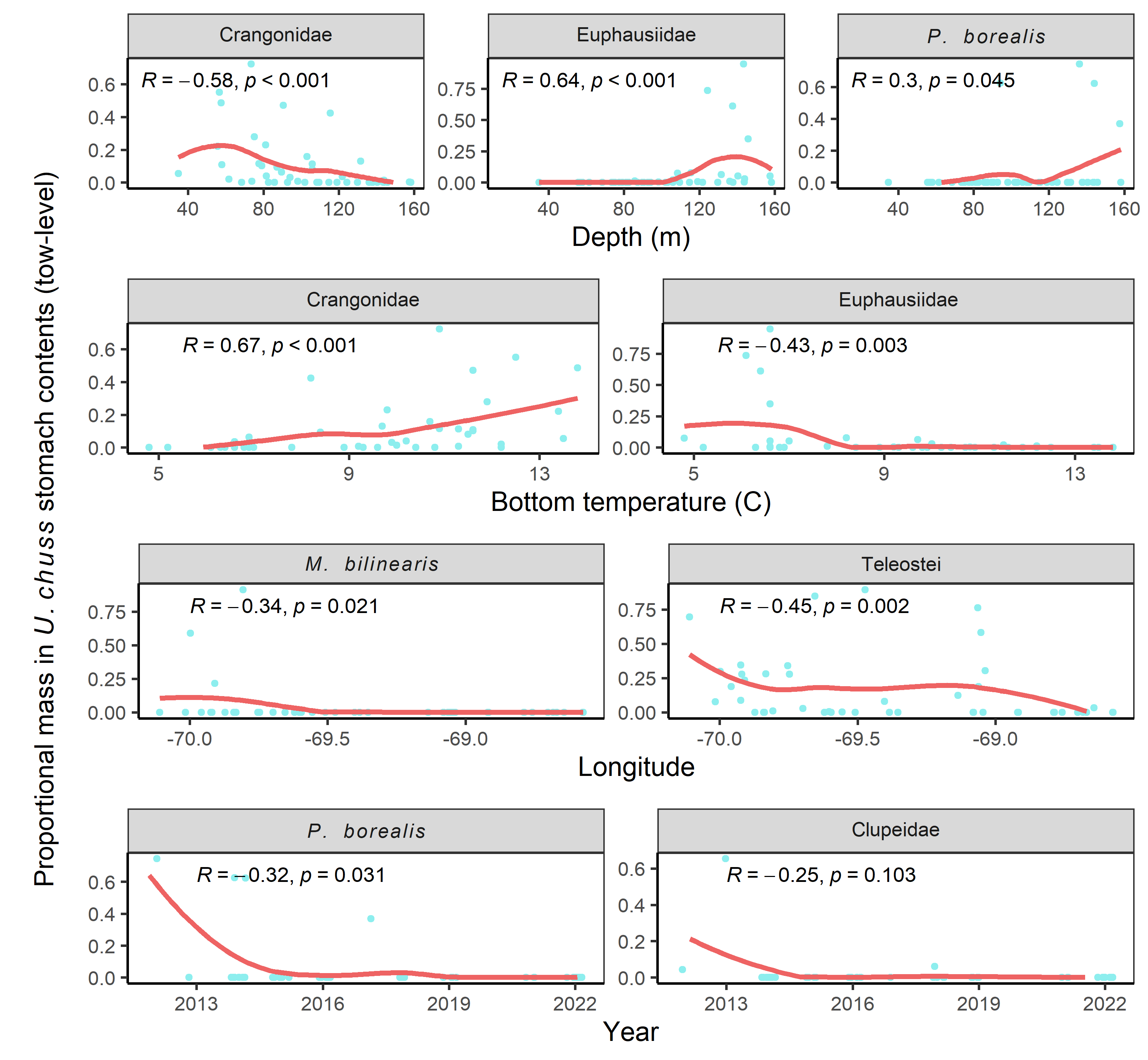


Figure S15. Scatter plots depicting relationships between tow-level proportional mass of prey in stomach contents of *Urophycis tenuis* and tow depth, bottom temperature, longitude and survey year. Results of Spearman rank correlation are displayed in each plot, and only prey taxa with a p-value <0.05 are included. Blue points represent individual tow-level values. Red lines show the fit of locally estimated scatterplot smoothing (LOESS). Statistical significance of a non-monotonic relationship should be considered a spurious correlation.


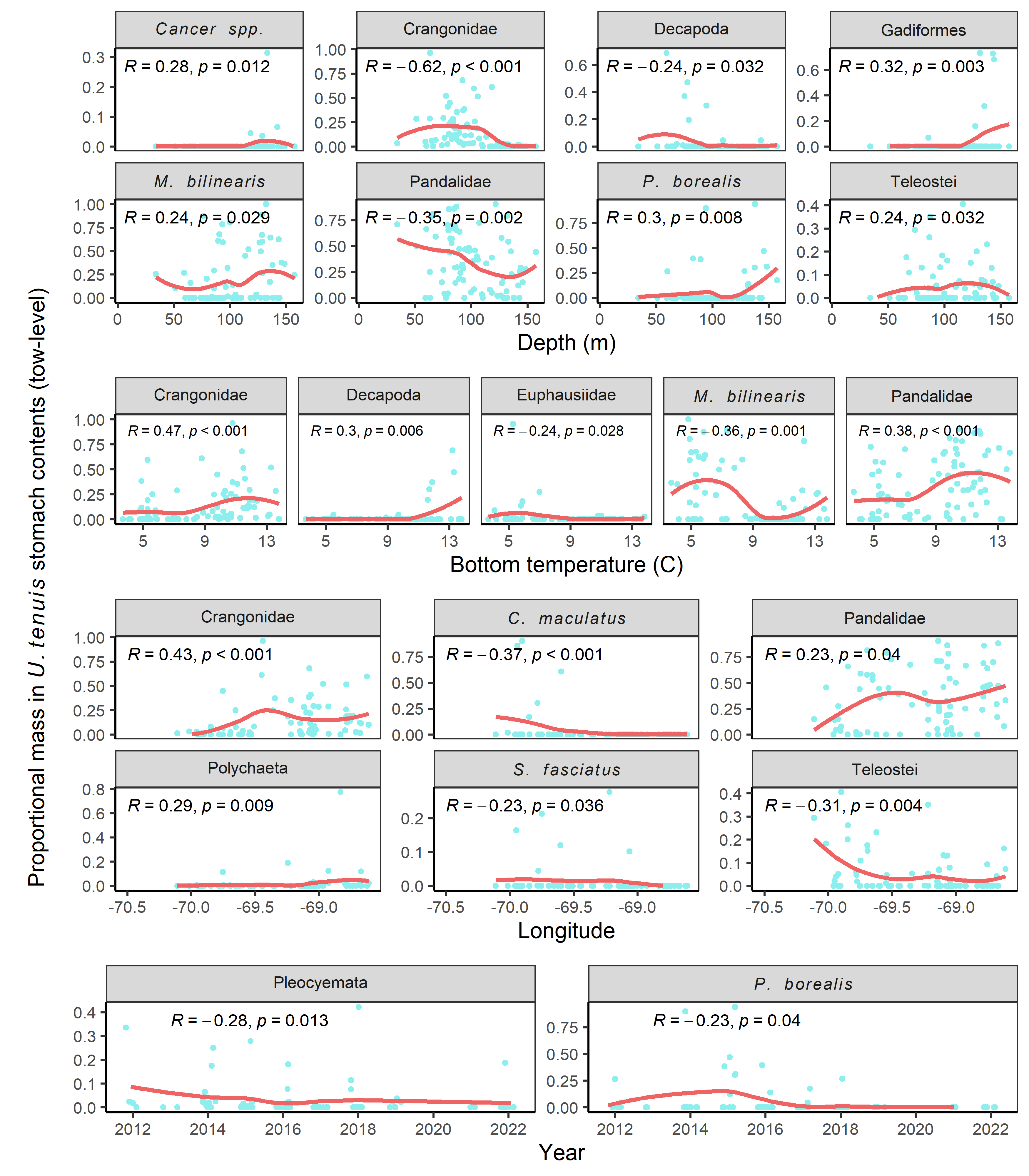

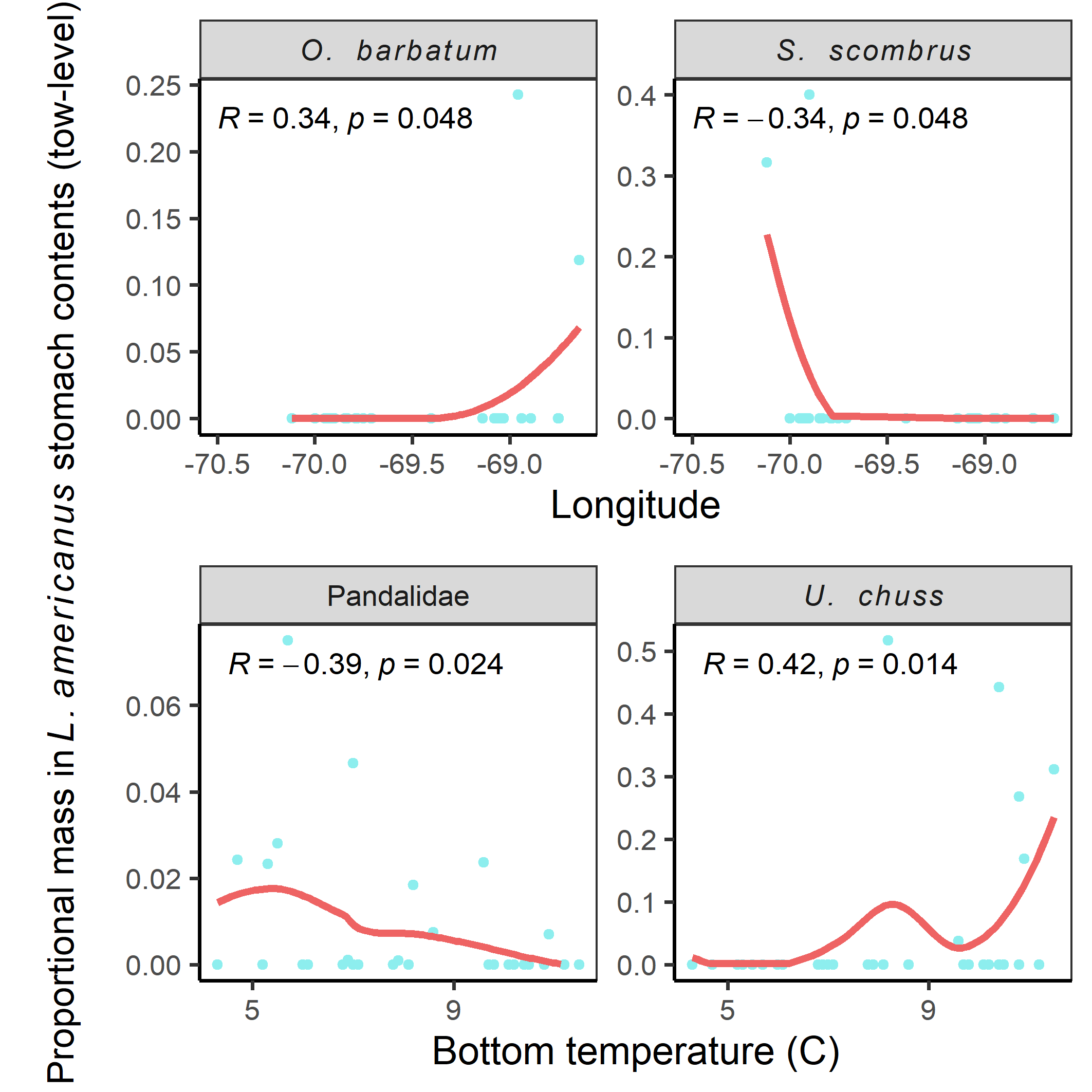


Figure S16. Scatter plots depicting relationships between tow-level proportional mass of prey in stomach contents of *Lophius americanus* and tow longitude and bottom temperature. Results of Spearman rank correlation are displayed in each plot, and only prey taxa with a p-value <0.05 are included; no correlations with depth or year were significant for *L. americanus*. Blue points represent individual tow-level values. Red lines show the fit of locally estimated scatterplot smoothing (LOESS). Statistical significance of a non-monotonic relationship should be considered a spurious correlation.

Table S5. Posterior point estimates of the mean coefficient, error, and 95% credible interval (CI) of parameters in Bayesian logistic regressions of prey occurrence in groundfish predator stomachs for five focal prey taxa (sub-tables A-E). Negative values are shown in red. In each model, *Merluccius bilinearis* is the reference predator and spring is the reference season for these categorical parameters. The number of observations for each model was 3,638, except in the Alosinae model (n = 3,586), which omits Atlantic cod (no occurrences). Posterior estimates for the effects of relative prey availability (modeled separately) are reported in the main text.

| **A)** Alosinae | | | | |
| --- | --- | --- | --- | --- |
|  | Mean | Error | 2.5% CI | 97.5% CI |
| Intercept | -4.50 | 0.51 | -5.53 | -3.55 |
| Predator Species (*Urophycis chuss*) | -2.40 | 1.25 | -5.40 | -0.51 |
| Predator Species (*Urophycis tenuis*) | -1.10 | 0.59 | -2.35 | -0.03 |
| Predator Species (*Lophius americanus*) | 0.91 | 0.40 | 0.12 | 1.68 |
| Predator Species (*Squalus acanthias*) | 1.02 | 0.68 | -0.28 | 2.35 |
| Predator Length | 0.55 | 0.15 | 0.24 | 0.84 |
| Season (Fall) | -0.77 | 0.33 | -1.41 | -0.12 |
| Depth | -0.65 | 0.16 | -0.97 | -0.34 |
| Longitude | -0.12 | 0.14 | -0.39 | 0.16 |
| Year | 0.06 | 0.06 | -0.07 | 0.19 |
|  |  |  |  |  |
| **B)** *Clupea harengus* | | | | |
|  | Mean | Error | 2.5% CI | 97.5% CI |
| Intercept | -1.71 | 0.27 | -2.25 | -1.19 |
| Predator Species (*Urophycis chuss*) | -2.46 | 0.64 | -3.88 | -1.38 |
| Predator Species (*Urophycis tenuis*) | -2.29 | 0.44 | -3.23 | -1.49 |
| Predator Species (*Lophius americanus*) | -1.12 | 0.38 | -1.90 | -0.41 |
| Predator Species (*Squalus acanthias*) | -1.34 | 0.69 | -2.70 | -0.01 |
| Predator Species (*Gadus morhua*) | -1.67 | 0.88 | -3.63 | -0.16 |
| Predator Length | 0.63 | 0.15 | 0.33 | 0.92 |
| Season (Fall) | -0.63 | 0.25 | -1.12 | -0.15 |
| Depth | -0.54 | 0.12 | -0.78 | -0.31 |
| Longitude | -0.13 | 0.11 | -0.34 | 0.08 |
| Year | -0.19 | 0.04 | -0.28 | -0.11 |
|  |  |  |  |  |
| **C)** *Merluccius bilinearis* | | | | |
|  | Mean | Error | 2.5% CI | 97.5% CI |
| Intercept | -0.63 | 0.15 | -0.91 | -0.34 |
| Predator Species (*Urophycis chuss*) | -1.68 | 0.22 | -2.13 | -1.26 |
| Predator Species (*Urophycis tenuis*) | -0.86 | 0.13 | -1.13 | -0.61 |
| Predator Species (*Lophius americanus*) | -0.67 | 0.16 | -0.98 | -0.36 |
| Predator Species (*Squalus acanthias*) | -4.42 | 0.85 | -6.32 | -2.96 |
| Predator Species (*Gadus morhua*) | -1.35 | 0.49 | -2.39 | -0.46 |
| Predator Length | 0.45 | 0.08 | 0.29 | 0.60 |
| Season (Fall) | -0.98 | 0.13 | -1.23 | -0.73 |
| Depth | 0.16 | 0.06 | 0.04 | 0.28 |
| Longitude | -0.22 | 0.05 | -0.32 | -0.11 |
| Year | -0.06 | 0.02 | -0.10 | -0.02 |

Table S5 continued

| **D)** Euphausiidae | | | | |
| --- | --- | --- | --- | --- |
|  | Mean | Error | 2.5% CI | 97.5% CI |
| Intercept | -1.20 | 0.17 | -1.53 | -0.88 |
| Predator Species (*Urophycis chuss*) | -0.09 | 0.15 | -0.39 | 0.21 |
| Predator Species (*Urophycis tenuis*) | -0.79 | 0.15 | -1.08 | -0.50 |
| Predator Species (*Lophius americanus*) | -3.15 | 0.48 | -4.18 | -2.30 |
| Predator Species (*Squalus acanthias*) | -0.35 | 0.57 | -1.51 | 0.73 |
| Predator Species (*Gadus morhua*) | -2.13 | 0.83 | -4.00 | -0.76 |
| Predator Length | -0.31 | 0.13 | -0.57 | -0.05 |
| Season (Fall) | -0.41 | 0.14 | -0.69 | -0.14 |
| Depth | 0.71 | 0.08 | 0.56 | 0.86 |
| Longitude | 0.02 | 0.06 | -0.10 | 0.13 |
| Year | -0.11 | 0.02 | -0.15 | -0.06 |
|  |  |  |  |  |
| **E)** *Pandalus borealis* | | | | |
|  | Mean | Error | 2.5% CI | 97.5% CI |
| Intercept | -3.30 | 0.36 | -4.03 | -2.63 |
| Predator Species (*Urophycis chuss*) | 0.47 | 0.40 | -0.30 | 1.25 |
| Predator Species (*Urophycis tenuis*) | 0.78 | 0.34 | 0.12 | 1.44 |
| Predator Species (*Lophius americanus*) | 1.25 | 0.36 | 0.54 | 1.96 |
| Predator Species (*Squalus acanthias*) | -1.56 | 1.42 | -4.87 | 0.74 |
| Predator Species (*Gadus morhua*) | 0.15 | 0.88 | -1.79 | 1.67 |
| Predator Length | 0.22 | 0.15 | -0.09 | 0.52 |
| Season (Fall) | 0.47 | 0.29 | -0.08 | 1.03 |
| Depth | 0.53 | 0.15 | 0.25 | 0.84 |
| Longitude | -0.18 | 0.13 | -0.43 | 0.06 |
| Year | -0.40 | 0.05 | -0.50 | -0.30 |
|  |  |  |  |  |
